# Interleukin-2 induces the in vitro maturation of human pluripotent stem cell-derived intestinal organoids

**DOI:** 10.1038/s41467-018-05450-8

**Published:** 2018-08-02

**Authors:** Kwang Bo Jung, Hana Lee, Ye Seul Son, Mi-Ok Lee, Young-Dae Kim, Soo Jin Oh, Ohman Kwon, Sunwha Cho, Hyun-Soo Cho, Dae-Soo Kim, Jung-Hwa Oh, Matthias Zilbauer, Jeong-Ki Min, Cho-Rok Jung, Janghwan Kim, Mi-Young Son

**Affiliations:** 1 Korea Research Institute of Bioscience and Biotechnology (KRIBB), Daejeon, 34141 Republic of Korea; 2 KRIBB School of Bioscience, Korea University of Science and Technology (UST), Daejeon, 34113 Republic of Korea; 3 Asan Institute for Life Sciences, Asan Medical Center & Department of Convergence medicine, College of Medicine, University of Ulsan, Seoul, 05505 Republic of Korea; 4 Korea Institute of Toxicology, Daejeon, 34114 Republic of Korea; 5 Department of Paediatric Gastroenterology, Hepatology and Nutrition, Cambridge University Hospitals, Addenbrooke’s, Cambridge Biomedical Campus, Hills Road, Cambridge, CB2 0QQ UK

## Abstract

Human pluripotent stem cell (hPSC)-derived intestinal organoids (hIOs) form 3D structures organized into crypt and villus domains, making them an excellent in vitro model system for studying human intestinal development and disease. However, hPSC-derived hIOs still require in vivo maturation to fully recapitulate adult intestine, with the mechanism of maturation remaining elusive. Here, we show that the co-culture with human T lymphocytes induce the in vitro maturation of hIOs, and identify STAT3-activating interleukin-2 (IL-2) as the major factor inducing maturation. hIOs exposed to IL-2 closely mimic the adult intestinal epithelium and have comparable expression levels of mature intestinal markers, as well as increased intestine-specific functional activities. Even after in vivo engraftment, in vitro-matured hIOs retain their maturation status. The results of our study demonstrate that STAT3 signaling can induce the maturation of hIOs in vitro, thereby circumventing the need for animal models and in vivo maturation.

## Abstract (web summary)

Human pluripotent stem cell-derived intestinal organoids (hIOs) are a useful model with which to study intestinal development and disease, but they require in vivo maturation to resemble adult tissue. Here, the authors show that T lymphocyte-derived IL-2 induces hIO maturation in vitro through the activation of STAT3.

## Introduction

The adult intestinal epithelium performs diverse physiological functions: as a major interface between the interior and exterior environment of an organism it is responsible for digestion and nutrient absorption, it presents a mucosal barrier to microorganisms, and regulates the immune response to pathogens. These functions are enacted by specialized cell types, including absorptive enterocytes and the secretory cell types known as goblet, enteroendocrine, and Paneth cells, which secrete mucin, hormones and anti-bacterial peptides, respectively^1^. In addition to the monolayer of intestinal epithelial cells, the complete intestine contains other cell types, such as fibroblasts, immune cells, interstitial cells, vascular and lymphatic endothelial cells, smooth muscle cells, and enteric neurons^2^, all of which interact extensively with each other during development to develop the cooperation necessary for full gut functionality in the adult^3^.

Recently, 3D multicellular intestinal organoids (hIOs) have been developed using either human pluripotent stem cells (hPSCs)^4^ or adult intestinal stem cells^5^ as the source material. hIOs contain crypt-like and villus-like structures, as well as all four major cell types of the small intestinal epithelium, so recapitulating the architecture and cellular diversity of the epithelium. In addition, hIOs exhibit basic physiological functions like the secretion of mucus and absorption of amino acids^4,6^. Once established, hIOs can be passaged in vitro multiple times for up to 1 year. In contrast to hIOs formed from adult stem cells, hPSC-derived hIOs are surrounded by a primitive mesenchyme which can differentiate into smooth muscle, myofibroblasts, and fibroblasts during the differentiation protocol, meaning that hPSC-derived hIOs simultaneously model both the epithelial and submucosal layers of the human intestine in vitro^3^. The elucidation of a stepwise differentiation protocol directing hPSCs towards a functional intestinal epithelium has thus in and of itself greatly improved our understanding of human intestinal development. However, despite the significant similarities in structure and function between hIOs and the intestine, hPSC-derived hIOs still retain immature characteristics, making them more similar to the fetal intestine^7,8^. These immature hIOs can further develop into functionally mature, adult-like small intestine, but only in vivo following transplantation into the kidney capsule or when grown as a teratoma in an immunocompromised mouse^9,10^. The mature small intestine has unique characteristics, including the expression of the mature stem cell marker, OLFM4, as well as an upregulated expression of genes required for digestion, transport, and gut immunity^11,12^. Currently, the mechanisms promoting the full maturation of hIO, including the identity of signaling cues, supporting cell types and the surrounding environment, are not known.

In this study, we demonstrate that interleukin-2 (IL-2)-secreting immune cells promote the maturation of hPSC-derived hIOs as part of an in vitro co-culture system. Further investigations demonstrated that the activation of STAT3 signaling was crucial for the in vitro maturation of hIOs. Following in vitro maturation, hIOs exhibited the characteristics of mature adult intestinal epithelium in terms of both gene expression profile and diverse functionality. Our findings thus shed light on the biological mechanism of neonatal gut development, underlining the importance of interactions between immune and epithelial cells for the maturation of the gut. The inclusion of the immune component into the stepwise differentiation protocol to form mature hIOs from hPSCs resolves a previous limitation of this technology, enabling the use of pre-established normal and induced hPSCs for studies of adult physiology and diseases of the intestine.

## Results

### Co-culture with T lymphocytes promotes hIO maturation

**Fig. 1:**
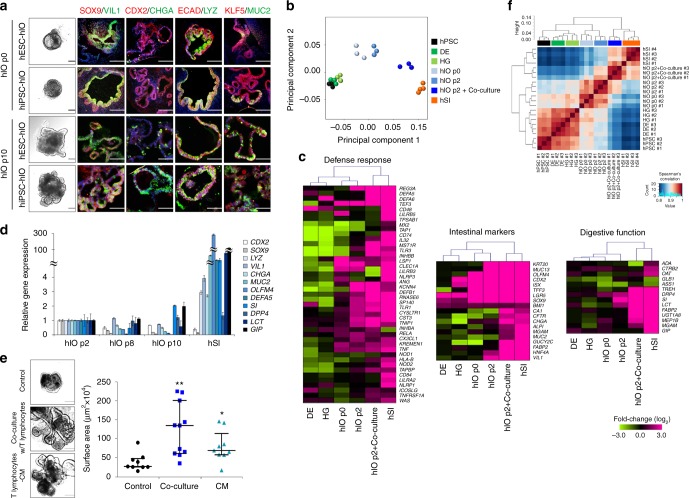
hPSC-derived hIOs undergo maturation during co-culture with immune cells. **a** Representative morphology and immunofluorescent staining of hIOs (p0, p10) for gut-specific markers (SOX9, CDX2, and KLF5); the enterocyte marker, VIL1; the goblet cell marker, MUC2; the Paneth cell marker, LYZ; the enteroendocrine cell marker, CHGA; and the epithelial marker, ECAD. Scale bar, 200 μm. **b** Principal component analysis (PCA) of differentially expressed genes (>2-fold change) from microarray experiments comparing definitive endoderm cells (DE) (*n* = 3), hindgut cells (HG) (*n* = 3), hIOs (p0) (*n* = 3), hIOs (p2) (*n* = 3), co-cultured hIOs (*n* = 3) and adult human small intestine (hSI) (*n* = 4). **c** Heatmaps of genes involved in the defense response, intestinal markers and genes required for digestion, demonstrating that co-cultured hIOs are the cell type most similar to adult hSI. **d** qPCR analysis of the expression of intestinal markers and maturation markers in hIO (p2, p8, p10) and hSI. **e** Representative images of morphological changes occurring in hIOs following co-culture with PMA/ionophore-stimulated Jurkat T cells or treatment with stimulated Jurkat T cell-conditioned medium (CM). Scale bar, 1 mm. Quantitative assessment of the size of hIOs after two passages; *n* = 9 (control), *n* = 11 (co-culture), *n* = 9 (CM) hIOs per group. PMA phorbol myristate acetate, CI calcium ionophore. **f** Spearman’s correlation was used to cluster samples and generate a heatmap. Red indicating the highest level of similarity between samples and blue indicates the lowest level of similarity. The dendrogram indicates that co-cultured hIOs are similar to hSI samples. Data are presented as mean values of replicates ± SEM. **p < 0.01, *p *<* 0.05, according to *t*-test

Three hIO lines were derived from hPSCs following a previously described^13,14^, stepwise hIO differentiation protocol: one hIO line was derived from a human embryonic stem cell (hESC) line and two lines were obtained from fully characterized, integration-free human induced pluripotent stem cell (hiPSC) lines reprogrammed from human fibroblasts (Supplementary Fig. 1). As expected, hPSCs could be efficiently differentiated into definitive endoderm, hindgut, and hIO fates with their accompanying characteristic morphologies and the expression of stage-specific markers (Supplementary Fig. 2a, b and Fig. 1a). During the first two passages we observed an increase in the expression of intestinal markers, such as intestinal transcription factors (*CDX2*, *SOX9*, and *ISX*) and cell type-specific markers, including *VIL1* (villin 1 for enterocytes), *CHGA* (chromogranin A for enteroendocrine cells), *LYZ* (lysozyme for Paneth cells), *MUC2* (mucin 2 for goblet cells), and the mesenchymal marker *VIM* (vimentin), confirming the specification of hPSCs for an intestinal identity (Supplementary Fig. 2c). A microarray-based principal component analysis (PCA) further confirmed the acquisition of intestinal identity by the hIOs over the first two passages (Fig. 1b). However, as previously reported^7^, when compared directly to adult human small intestine (hSI), hIOs at passage 2 did not fully recapitulate the expression level of genes such as *OLFM4*, an intestinal stem cell (ISC) marker, or a panel of genes expressed by Paneth cells that are responsible for host defense and digestive function (Fig. 1c and Supplementary Table 1). Additional passages (up to 10 passages over 150 days) did not result in any further maturation of hIOs, as assessed by the gene expression levels of a panel of intestinal maturation markers, such as *OLFM4*, *DEFA5*, *SI*, *DPP4*, *LCT*, and *GIP* (Fig. 1d).

In adults, the intestinal epithelium interacts with immune cells in the intestinal mucosa to maintain intestinal homeostasis and mucosal immunity^15,16^. We hypothesized that immune system components, such as T lymphocytes, present in the intestinal epithelium and lamina propria^17^, could play a role in the maturation of the intestinal epithelium, and so we adapted our hIO culture system to co-culture with immune cells, enabling crosstalk between the hIOs and the immune cells via the soluble factors secreted from each cell. Various cytokines and chemokines were highly expressed in phorbol myristate acetate (PMA)/ionophore-stimulated Jurkat T cells compared to non-stimulated Jurkat T cells (Supplementary Fig. 3a, b). Therefore, hIOs were placed onto Transwell inserts and introduced into 12-well plates together with stimulated Jurkat T cells (Supplementary Fig. 4a). The co-culture of hIOs and stimulated Jurkat T cells led to a significant increase in organoid size, as well as to an increase in the number of budding structures present on each organoid, compared to the co-culture with non-stimulated Jurkat T cells (Fig. 1e and Supplementary Fig. 4b, c). hIOs cultured in the presence of conditioned medium (CM) taken from Jurkat T cells also showed similar changes in morphology (Fig. 1e), suggesting the involvement of secreted paracrine factors. Using Spearman’s correlation analysis, we observed that the expression profile of co-cultured hIOs is most closely related to that of hSI (Fig. 1f). This finding was further supported by data obtained in a microarray analysis of intestinal markers and genes involved in the defense response and digestion (Fig. 1c).

### IL-2/STAT3 signaling is required for hIO maturation

**Fig. 2:**
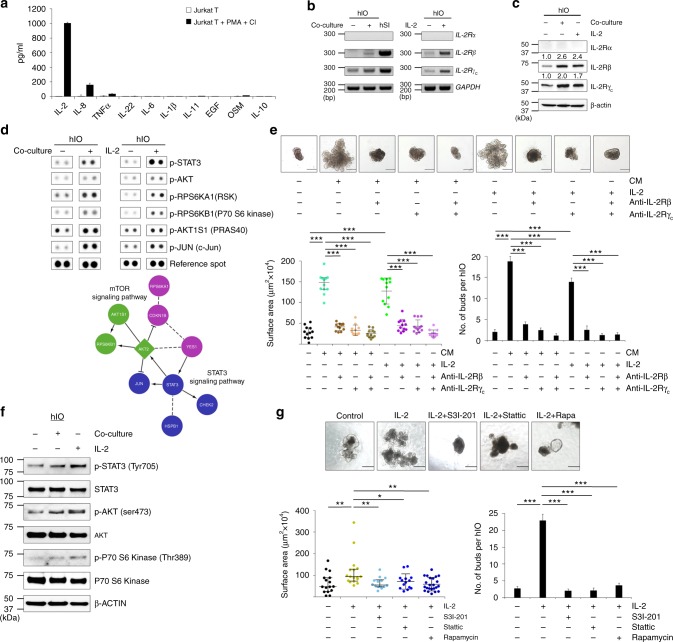
IL-2 activates STAT3 pathway to induce the in vitro maturation of hIOs. **a** ELISA quantification of IL-2, IL-8, TNFα, IL-22, IL-6, IL-1β, IL-11, EGF, OSM, and IL-10 concentrations in the culture supernatant of stimulated and unstimulated Jurkat T cells. Expression of IL-2R subunits as analyzed by RT-PCR **b** and Western blot analyses **c** in co-cultured or IL-2-treated hIOs. **d** Phosphorylation levels of proteins from multiple signaling pathways in control, co-cultured and IL-2-treated hIOs as reported by a human phospho-kinase array (upper panels). Analysis by functional interaction (FI) network highlighted a significant enrichment of phospho-proteins in the mTOR and STAT3 signaling pathways (lower panels). In the FI network, arrows represent activating/catalyzing connections, solid lines ending in a perpendicular line represent inhibition, solid lines represent complexes or inputs and dashed lines represent predicted FI connections. **e** Representative images of the morphology of hIOs cultured in the presence of 1 ng/ml IL-2, a key component of the co-culture system, or stimulated Jurkat T conditioned medium (CM) with or without IL-2R-inactivating antibodies (anti-IL-2Rβ, anti-IL-2Rγ_c_) for two passages. Quantitative assessment of the size of hIOs (left bottom) and the number of budding structure per hIO (right bottom); *n* = 12 hIOs per group. **f** Representative Western blot analysis of p-STAT3, p-AKT and p-P70 S6 Kinase levels in co-cultured and IL-2-treated hIOs. **g** hIOs cultured in the presence of IL-2 (1 ng/ml) with or without the addition of S3I-201 (5 μM), Stattic (1 μM) or Rapamycin (Rapa; 10 nM). Quantitative assessment of the size of hIOs after one passage (14 days) (left bottom) and the number of budding structure per hIO (right bottom); *n* = 14 hIOs per group. Data are presented as mean values of replicates ± SEM. ***p < 0.001, **p < 0.01, and *p *<* 0.05 according to *t*-test. Scale bar, 500 μm

Next, we assessed the levels of several cytokines in the CM of PMA/ionophore-stimulated Jurkat T cells to identify the major secreted factor in this co-culture system. Compared to unstimulated cells, stimulated Jurkat T cells released significantly higher amounts of IL-2 than other cytokines, including tumor necrosis factor alpha (TNFα), IL-8, IL-22, IL-6, IL-1β, IL-11, EGF, OSM, and IL-10 (Fig. 2a and Supplementary Table 2). Interestingly, the corresponding IL-2 receptor (IL-2R) complex containing IL-2 receptor beta (IL-2Rβ) and gamma (IL-2Rγ_c_) chains was expressed in the hIOs and the levels of gene and protein expression of receptor complex were increased upon co-culture (Fig. 2b, c). To investigate the signaling profile more thoroughly, we performed a phospho-kinase array analysis. Of the 43 kinases included in the array, 21 kinases exhibited a noticeable difference (>1.2-fold change) in their phosphorylation status when comparing the control and co-culture systems (Supplementary Fig. 5a, b). Pathway enrichment analysis revealed that the IL-2-mediated signaling pathway was one of the most heavily upregulated pathways in the co-cultured hIOs (adjusted FDR < 0.001; Supplementary Fig. 5c).

Having identified IL-2 as a candidate factor for promoting the in vitro maturation of hIOs, we next applied recombinant human IL-2 (rhIL-2) to our hIOs. The previously identified pattern of phospho-activation could be recapitulated solely by the use of IL-2 rather than Jurkat T cells, as assessed by the activation of previously identified downstream effectors such as STAT3 and c-Jun (Fig. 2d and Supplementary Fig. 5d, e). Treatment with IL-2 at a concentration of 1–8 ng/ml was sufficient to significantly increase organoid size (Supplementary Fig. 6a). Further, hIOs cultured with IL-2 significantly increased in size and the average number of buds per hIO similar to those with CM of PMA/ionophore-stimulated Jurkat T cells. This increase was completely inhibited when the selective blockade of the IL-2 receptor was induced by a combination of antibodies against IL-2Rβ and IL-2Rγ_c_ (Fig. 2e)._._ Given that hIOs that have already been matured in vitro by IL-2 treatment did not require a continuous supply of IL-2 (Supplementary Fig. 7a–c), this in vitro maturation status of hIOs did not appear to be a transient.

In the phospho-kinase array analysis, phosphorylation of STAT3 (Y705) was the most substantially altered (3.3-fold increase) upon IL-2 treatment (Supplementary Fig. 5d, e), followed by the phosphorylation of AKT and P70 S6 Kinase, implying the involvement of the STAT3 and mTOR signaling pathways in hIO maturation (Supplementary Fig. 5f and Fig. 2d,f). In support of this conclusion, the effect of IL-2 on hIO growth was prevented by the addition of the specific small molecule inhibitors of STAT3, S3I-201 or Stattic, or of mTOR, rapamycin (Fig. 2g). We also found that treatment with STAT3 activators, such as Colivelin or IL-22, promoted the growth of hIOs with many crypt-like budding structures (Supplementary Fig. 8a, b). These data suggest that the effect of IL-2 on the morphology of hIOs is mediated by the activation of STAT3 signaling.

### In vitro-matured hIOs more closely resemble the adult hSI

**Fig. 3:**
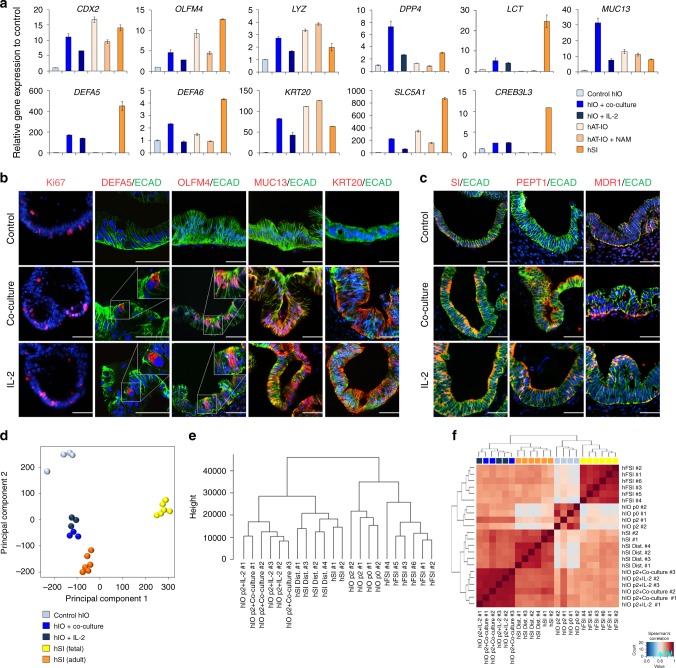
Co-cultured or IL-2-treated hIOs express the markers of mature small intestine. **a** qPCR analysis of the expression of markers of intestinal maturation in control, co-cultured and IL-2-treated hIOs, human adult tissue-derived intestinal organoids (hAT-IOs) cultured in the presence or absence of nicotinamide (NAM), and hSI. NAM was used to improve the culture efficiency of hAT-IOs. Fold changes in expression level are relative to control hIOs. **b** Immunofluorescent staining of control, co-cultured and IL-2-treated hIOs with a proliferation marker (Ki-67), an epithelial marker (ECAD) and mature intestinal markers (DEFA5, OLFM4, MUC13, and KRT20). Scale bar, 50 μm. **c** Immunofluorescent staining for the intestinal enzyme sucrase-isomaltase (SI) and intestinal transporters (peptide transporter 1, PEPT1; multidrug resistance protein 1, MDR1). Scale bar, 50 μm. **d** PCA of the RNA-sequencing datasets for control hIOs (*n* = 4), co-cultured hIOs (hIO + co-culture, *n* = 3), IL-2 treated hIOs (hIO + IL-2, *n* = 3), human fetal small intestine (hFSI) (*n* = 6) and adult human small intestine (hSI) (*n* = 6). **e** A dendrogram based on hierarchical clustering of the gene sets from the RNA-sequencing using Canberra distance. Branch lengths indicate the degree of difference between samples. **f** Spearman’s correlation was used to cluster samples and generate a heatmap. Red indicates the highest level of similarity between samples and blue indicates the lowest level of similarity. Data are presented as mean values of replicates ± SEM

We sought to further characterize the effect of IL-2 on the in vitro maturation of hIOs by performing global gene expression profiling using microarray to compare untreated hIOs, hIOs in co-culture with stimulated Jurkat T cells, hIOs treated with IL-2 and hSI (Supplementary Fig. 9a). Co-culture of hIOs with stimulated Jurkat T cells was slightly more effective than the treatment of hIOs with IL-2 in recapitulating the profile of hSI (55.9% of genes shifted towards the profile of hSI vs 49.8% of genes shifted, respectively). Statistical analyses, including PCA, hierarchical clustering with the Canberra distance, and Spearman’s correlation, revealed strong correlations between the expression profiles of the co-cultured or IL-2-treated hIOs and the hSI (Supplementary Fig. 9b–d). Gene Ontology (GO) enrichment analysis on differentially expressed genes involved in intestinal maturation after the co-culture or IL-2 treatment of hIOs revealed a significant up-regulation of genes annotated with GO terms related to cellular response to stimulus, defense response, regulation of immune system process, and response to cytokines (Supplementary Fig. 9e and Supplementary Table 1). Quantitative RT-PCR (qPCR) analysis was performed for a number of mature intestinal markers: *CDX2*, an intestine-specific marker; *OLFM4*, an ISC marker of the mature intestine; the Paneth cell markers, *DEFA5*, *DEFA6*, and *LYZ*; and the mature intestinal differentiation markers *KRT20*, *MUC13*, *SLC5A1*, *CREB3L3*, *DPP4*, and *LCT* (Fig. 3a). For most genes, the level of expression was highly upregulated compared to control hIOs and was similar or at least comparable to hSI. Interestingly, hPSC-derived hIOs further matured in vitro by co-culture or by treatment with IL-2 showed a similar level of maturation to human adult tissue-derived intestinal organoids (hAT-IOs; Fig. 3a). Immunofluorescence consistently detected the expression of the DEFA5, OLFM4, MUC13, and KRT20 proteins only in hIOs matured in vitro by co-culture or treatment with IL-2 and not in the control hIOs (Fig. 3b). In addition, functional brush-border enzymes and intestinal transporters, such as sucrase-isomaltase (SI), MDR1 and peptide transporter 1 (PEPT1), were exclusively expressed in the co-cultured or IL-2-treated hIOs (Fig. 3c). The expression of mature intestinal markers was completely inhibited upon blocking of IL-2 signaling (Supplementary Fig. 6b). To assess the maturation status of hIOs based on global gene expression patterns, we conducted PCA (Fig. 3d), hierarchical clustering of with the Canberra distance (Fig. 3e), and Spearman’s correlation (Fig. 3f) analyses using our original and publicly available RNA-sequencing datasets of human fetal and adult small intestine samples (Supplementary Table 3). These analyses consistently demonstrated that the transcriptomes of the in vitro-matured hIOs co-cultured or treated with IL-2 more closely resembled the profile of the hSI than that of fetal small intestines.

### hIOs matured in vitro display enhanced functionality

**Fig. 4:**
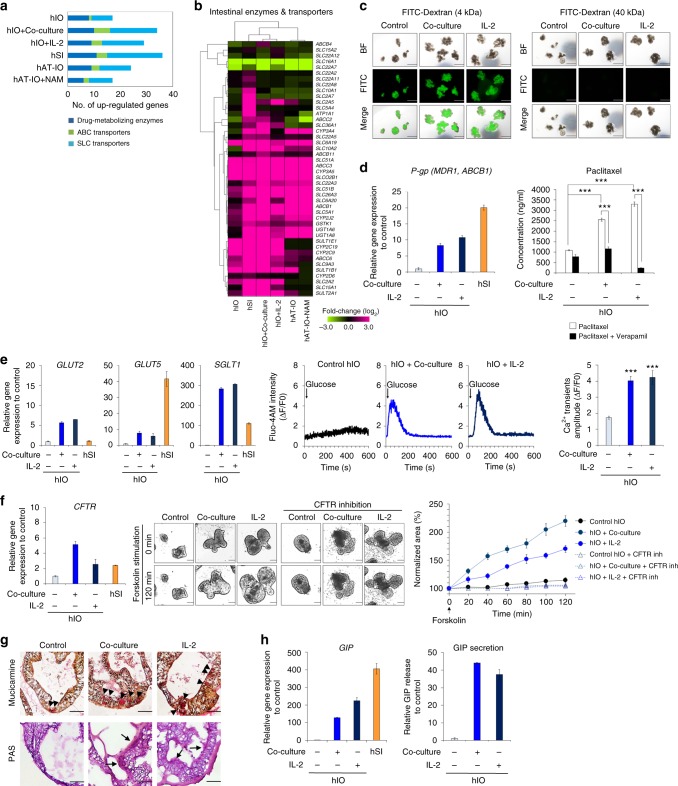
In vitro-matured hIOs have significantly enhanced functionalities. **a** Comparison of the gene expression of drug-metabolizing enzymes (phase I/II enzymes) and intestinal transporters (ABC transporters and SLC transporters) among hIOs, hAT-IOs, and hSI. The graph represents the number of genes with expression >2-fold when compared with the control hIOs. **b** Hierarchical clustering of the expression level of genes encoding intestinal enzymes and transporters in control, co-cultured, and IL-2-treated hIOs, hAT-IOs, and hSI. **c** Paracellular permeability of hIOs incubated with 4 kDa FITC-Dextran and 40 kDa FITC-Dextran. Scale bar, 1 mm. **d** Expression levels of the transporter *P-gp* (*MDR1*, *ABCB1*) relative to control hIOs as assessed by qPCR (left panel). Apical concentration of paclitaxel following 2 h incubation in the absence or presence of verapamil in control, co-cultured or IL-2-treated hIOs (right panel, *n* = 20 per group). **e** Expression levels of the glucose transporters (*GLUT2*, *GLUT5*, *SGLT1*) in control, co-cultured, IL-2 treated hIOs, and hSI as assessed by qPCR (left panels). Glucose-induced Ca^2+^ transients in real-time manner in control, co-cultured and IL-2 treated hIOs (middle panels). Mean values of peak fluorescence intensity by using Fluo-4-AM calcium indicator (right panel) (*n* *=* 15 per group). **f** Expression levels of the *CFTR* in control, co-cultured, IL-2 treated hIO and hSI as assessed by qPCR (left panel). Representative images of the morphological changes of hIOs after treatment with forskolin (middle panel). Scale bar, 200 μm. Normalized forskolin-induced swelling of control, co-cultured, and IL-2 treated hIOs with or without CFTR inhibitors (GlyH101 + CFTR_inh_ 172) (right panel, *n* *=* 4 per group). **g** Mucicarmine staining for secreted mucin and acid mucopolysaccharide (black arrowheads), and PAS staining for the mucous layer (black arrows) and mucous producing goblet cells. Scale bar, 50 μm. **h** Expression levels of intestinal hormone *GIP* of control, co-cultured, and IL-2 treated hIOs and hSI (left panel). The levels of secreted GIP from enteroendocrine cells in hIOs ELISA for detection of, and normalized to DNA content (right panel). Data are presented as mean values of replicates ± SEM. ***p *<* 0.001 according to *t*-test

Because the mature hSI plays a major role in drug absorption and metabolism and expresses a diverse array of transporters and drug-metabolizing enzymes^18^, we compared the expression patterns of various transporters and metabolizing enzymes between in vitro-matured hIOs and hSI. The main intestinal cytochrome p450 enzymes, conjugation enzymes and transporters of the solute carrier (SLC) family (uptake), and ATP-binding cassette (ABC) family (efflux) were highly expressed in hSI, as expected (Fig. 4a). Of the 40 analyzed enzymes and transporters, 27 (67.5 %) and 25 (62.5 %) were upregulated by at least 2-fold in the hIOs matured by co-culture and treatment with IL-2, respectively; a greater number than is seen in hAT-IOs (Fig. 4b). Next, we tested the intestinal cell type-specific activity in the in vitro-matured hIOs, such as the efficacy of transport and secretion function by performing the various functional assays. hIOs have a functionally permeable intestinal epithelial barrier, as indicated by the paracellular diffusion pattern of FITC-dextran (Fig. 4c). P-glycoprotein (P-gp/MDR1/ABCB1) is a major efflux transporter that affects the pharmacokinetics of a wide range of drugs and xenobiotics^19^, and high P-gp expression was detected in the hIOs matured in vitro by either co-culture or treatment with IL-2 (Fig. 4d). To directly assess the activity of P-gp, paclitaxel, a prototypic substrate, was loaded to the basolateral side (outside) of hIOs incubated under control and in vitro-matured conditions. Following a 2 h incubation, the concentrations of paclitaxel at the apical side (inside of hIOs) were increased approximately 2.3-fold (*p* *<* 0.001) in the hIOs matured by co-culture and 3-fold (*p* *<* 0.001) in the hIOs matured by treatment with IL-2 when compared to concentrations at the apical side in control hIOs (Fig. 4d). The inhibition of P-gp activity by the addition of verapamil, a calcium channel blocker, abrogated this increased level of paclitaxel transport (Fig. 4d). Upon glucose stimulation, more intracellular Ca^2+^ was released from the endoplasmic reticulum (ER) in the co-cultured and IL-2-treated hIOs, which also exhibited higher Ca^2+^ transient amplitudes (ΔF/F0 based on the calcium indicator Fluo-4 AM) than were observed in the control hIOs (Fig. 4e). These results are consistent with the high expression level of the major glucose transporters of the intestine, such as *GLUT2* (*SLC2A2*), *GLUT5* (*SLC2A5*), and *SGLT1* (*SLC5A1*) (Fig. 4e), suggesting that the in vitro-matured hIOs contain more glucose-responsive mature enterocytes. The expression of cystic fibrosis transmembrane conductance regulator (CFTR) was significantly higher in the co-cultured and IL-2-treated hIOs (Fig. 4f). The results of forskolin-induced swelling (FIS) assays, which were used to measure the functional activity of the CFTR anion channel^20^, verified these findings. We quantified FIS of hIOs using real-time imaging microscopy and calculated the total area of hIOs for each time point after the forskolin induction. Upon stimulation with forskolin, more swelling was observed in the co-cultured and IL-2-treated hIOs than in the control hIOs, and FIS was blocked by the CFTR-specific inhibitors CFTR_inh_-172 and GlyH-101 in both co-cultured and IL-2-treated hIOs (Fig. 4f). Mucin and mucin secreting goblet cells were identified more often in the co-cultured or IL-2-treated hIOs compared to control hIOs by staining with mucicarmine and periodic acid-Schiff (PAS) (Fig. 4g). We also examined the secreted level of gastric inhibitory polypeptide (GIP), one of the hormones produced by functionally matured enteroendocrine cells^21^. These levels were significantly higher in the co-cultured or IL-2-treated hIOs than in the control hIOs, consistent with the observed up-regulation of *GIP* expression (Fig. 4h). Together, these data indicate that the in vitro-matured hIOs not only shifted their gene expression profiles towards that of mature hSI, but also contained matured intestinal-specific cell types with the appropriate physiological functionalities.

### In vitro-matured hIOs retain maturation markers in vivo

**Fig. 5:**
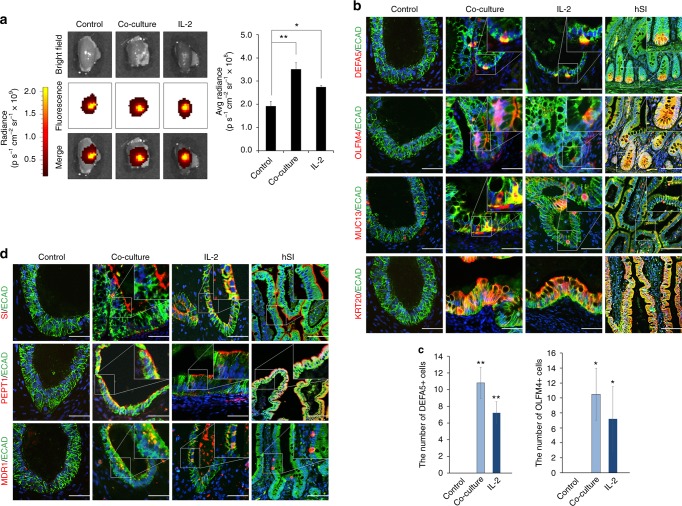
In vitro-matured hIOs can retain their maturation status in vivo. **a** Ex vivo fluorescence images of the kidney of immunodeficient (NSG) mice transplanted with DiR-labeled hIOs 1 week after transplantation using an IVIS imaging system (left panels). Quantification of the fluorescence intensity, expressed as the average radiance of the DiR-labeled hIOs (right panels; *n* = 3). p s^−1^ cm^−2^ sr^−1^, photons per second per cm^2^ per steradian. **b** Immunofluorescent staining for the intestinal maturation markers DEFA5, OLFM4, KRT20 and MUC13 in control, co-cultured and IL-2-treated hIOs following transplantation. Adult human small intestine (hSI) tissues were used as controls. **c** The number of DEFA5^+^ and OLFM4^+^ cells in control, co-cultured and IL-2-treated hIOs. Expression of DEFA5^+^ cells (left panel; *n* = 5) and OLFM4^+^ cells (right panel; *n* = 4). **d** Immunofluorescent staining for the intestinal enzyme sucrase-isomaltase (SI) and intestinal transporters (peptide transporter 1, PEPT1; multidrug resistance protein 1, MDR1) in control, co-cultured and IL-2-treated hIOs following transplantation. hSI tissues were used as controls. Data are presented as mean values of replicates ± SEM. *p *<* 0.05 and **p *<* 0.01 according to *t*-test

To assess the suitability of in vitro-matured hIOs for transplantation, mature hIOs were injected under the kidney capsule of immunodeficient NOD-SCID IL-2Rγ_c_^null^ (NSG) mice. The near-infrared fluorescent lipophilic dye, DiR, was used to label hIOs to allow their quantitative detection in vivo. The transplanted hIOs were confined inside of the kidney at 1 day post-transplantation, as detected by DiR fluorescence intensity (Supplementary Fig. 10a). The fluorescent signal was still detectable 1 week after transplantation (Fig. 5a). Analysis of the transplanted hIOs both by fluorescent signal and by histological examination revealed that only the hIOs matured in vitro by co-culture and by treatment with IL-2 maintained the increased sizes after the transplantation (Fig. 5a and Supplementary 10b). Intestinal cell type-specific markers were detected in all hIOs (Supplementary Fig. 10c), whilst markers of intestinal maturation were evident only in the transplants of in vitro-matured hIOs (Fig. 5b). DEFA5 and OLFM4 expression was exclusively observed in the co-cultured and IL-2-treated hIOs and confirmed by co-staining with Paneth cell marker (LYZ) and intestinal stem cell marker (ASCL2), respectively, and RNA fluorescence in situ hybridization (FISH) (Supplementary Fig. 11a, b). Quantitative analysis also showed that there were significantly more DEFA5- and OLFM4-positive cells in the in vitro-matured hIOs than in the control hIOs (Fig. 5c). In addition, a brush-border enzyme, SI, and the intestinal transporters MDR1 and PEPT1 were expressed only in the in vitro-matured hIOs after short-term transplantation (Fig. 5d). These results suggest that hIOs matured in vitro can retain their maturation status in vivo because they express the intestinal maturation markers even after they are transplanted. Given that the in vitro-matured hIOs maintained normal karyotypes (Supplementary Fig. 12), we suggest that they may represent an effective alternative source of cells for in vivo applications.

## Discussion

At present, it is possible to generate hIOs from two cell sources: hPSCs and adult intestinal tissue. Although hAT-IOs are a well-established technology and readily generate mature intestinal epithelium from surgically resected samples or endoscopic biopsies, they have some limitations. Firstly, genetically engineering of hAT-IOs is complex and inefficient, as it has to be performed within the context of 3D culture. Secondly, hAT-IO culture generates only the intestinal epithelium without the attendant mesenchymal components, in stark contrast to hPSC-derived hIOs. As whole genome-scale genetic engineering in embryonic and induced hPSCs is comparatively simple, the ability to generate fully mature, functional hIOs from hPSCs would allow the combined use of these complementary technologies. hPSC-derived hIOs therefore exhibit great potential for further studies of human gut development, physiology, and disease, as well as providing a readily accessible cell source for transplantation. However, generating fully functionally mature hIOs from hPSCs has until now proven to be technically challenging without the use of in vivo maturation^3,7,10^, and exposing hIOs to a xenobiological environment for their maturation precludes their use in clinical applications. Therefore, understanding the mechanism of hIO maturation has been of great interest to recapitulate this under conditions that would allow their clinical use.

Although various cell types are involved in the maturation and maintenance of intestinal tissue in vivo, the main players are cells in the intestinal epithelial lining and immune system^22^. Here we report that the co-culture with T lymphocytes or the introduction of an immune component, such as IL-2, to hPSC-derived hIO culture is key for the in vitro maturation of hIOs. Our in vitro maturation system of the intestine was constructed using hIOs comprising both intestinal epithelial cells (IECs) and surrounding stromal mesenchyme in combination with immune cells using a layer-by-layer approach, thus recapitulating epithelial-stromal-immune interactions. These cells are constantly communicating with each other and are influenced by interactions with soluble factors secreted by neighboring immune cells, which can maintain intestinal homeostasis and integrity^1,23^. Immune cells from the lamina propria and intraepithelial lymphocytes express various cytokines, such as IL-1, IL-2, IL-6, IL-10, and TNFα, which are known to stimulate proliferation and differentiation in IECs^24,25^. Actually, we used multicolor FACS analysis to demonstrate that more than half of the CD3^+^CD4^+^ and CD3^+^CD8^+^ T cell populations in the isolated mouse intestinal lamina propria secreted IL-2 (Supplementary Fig. 13a–e). Consistent with this observation, IL-2 was first identified as a major component in our in vitro maturation system. Recently, IL-2 was reported to contribute not only to modulating the growth and differentiation of immunocytes during inflammatory response but also to the maintenance and repair of intestinal mucosa and the epithelium^26,27^. Functional IL-2 receptors were endogenously expressed in isolated primary human IECs and human IEC lines, including Caco-2, HT-29, and T-84 cells, thus implying a physiological role for IL-2 in regulating epithelial functions^28,29^. IL-2-deficient mice develop a progressive inflammatory bowel disease closely resembling human ulcerative colitis (UC)^30,31^, and UC patients also exhibit a significant reduction in the expression of IL-2^32^. These results suggest that IL-2 may be important for the control of the intestinal structure and mucosal immunity.

Our results indicate that rhIL-2 induced the in vitro maturation of hIOs by binding to IL-2 receptors located on the surface of cells in the hIOs, subsequently activating the STAT3 and mTOR pathways. The in vitro maturation activity was abrogated by selectively blocking the IL-2 receptors on the hIOs or treatment with specific inhibitors for STAT3 and mTOR. IL-2 receptor specificity for intestinal maturation was also assessed using murine IL-2 and the murine intestinal organoid (mIO) system from IL-2 receptor gamma chain disrupted NSG mice (Supplementary Fig. 14a, b). However, more studies in the mIO system are required. This result is consistent with the increased IEC proliferation and regeneration via STAT3 after stimulation with IL-22^33,34^ and a very recent study demonstrating the role of mTOR signaling in promoting a proper IEC structure and function^35,36^. To our knowledge, this is the first report demonstrating the IL-2-mediated activation of the STAT3 and mTOR pathways in intestinal maturation and its functional significance. Although our in vitro data demonstrated that IL-2, as a mediator of the immune system, promotes intestinal maturation, additional in vivo experiments are necessary to clarify the mechanisms involved.

Our results indicate that the in vitro-matured hIOs acquired the morphological, molecular, and physiological hallmarks of mature hSI. We are the first to demonstrate that the transcriptome of in vitro-matured hIOs more closely resembles that of the hSI than that of the fetal small intestine. Both co-culture with human T lymphocytes and treatment with IL-2 significantly enhanced the expression of Paneth cell-specific genes and previously described intestinal maturation markers, which were expressed only after in vivo exposure^7,10^. Our in vitro-matured hIOs exhibit comparable levels of expression of various transporters and metabolizing enzymes to the hSI. Surprisingly, hAT-IOs exhibited lower expression levels of major drug transporters and metabolizing enzymes than were observed in the in vitro-matured hIOs. This result was attributed to the regional distribution of these transporters and enzymes in hAT-IOs derived from the human ileum, given that the expression of these genes is reduced in the ileum than in the upper small intestine^37^. Tissue-specific cells that are already differentiated undergo changes to achieve their specific functionality through the maturation process^38,39^. Generally, generating functional mature cells from hPSCs is technically challenging, ultimately resulting in hPSC-derived organoids of a less mature state^40^. However, we not only observed an extensive shift in the transcriptome profile in the in vitro-matured hIOs, but also showed the achievement of diverse functionality of the in vitro-matured hIOs, by performing various functional assays, including P-gp activity assay, glucose-stimulated intracellular calcium response to assess glucose transporter activity, FIS assay to assess CFTR activity, mucicarmine, and PAS staining to detect mucus producing mature goblet cells, and ELISA for GIP secreted from mature enteroendocrine cells. These findings are consistent with recent reports that the maturation status influences cell type-specific functions^41,42^. Therefore, the in vitro maturation method for hPSC-derived hIOs provided in this study not only induce further differentiation, but also promote maturation by achieving intestinal cell type-specific functionality. Although the intestinal epithelial cell diversity and the appropriate physiological functionalities of the hSI are recapitulated in the in vitro-matured hIOs, we believe that further studies will be required because complex organ-like structures, such as the spatial relationships of the crypt-villus axis, are not completely preserved.

Functional vessel formation and connection to the host vasculature are critical factors for successful engraftment^43^. Previous studies have demonstrated that when hIOs derived from hPSCs are transplanted underneath the mouse kidney capsule, they differentiate into mature adult cell types in vivo within 6–8 weeks^10^. Unfortunately, few vascular endothelial cells originating from the hPSCs were identified in the engraftment, and newly formed vessels failed to connect the vasculature of the transplanted parenchymal tissue to that of the host. However, we observed that the in vitro-matured hIOs in our system were able to successfully induce neovascularization in host blood vessels only 1 week after transplantation, even though no CD31-positive cells were detected before the hIOs were transplanted (Supplementary Fig. 10d–f). Although the mechanisms underlying the process by which the co-cultured or IL-2-treated hIOs induced neovascularization are not fully understood, the most likely explanation is that the human vascular endothelial cells that connected to the host vasculature were derived from the mesenchyme adjacent to the co-cultured and IL-2-treated hIOs. Consistent with this notion, we found that there were more laminated human mesenchyme (α-smooth muscle actin (α-SMA)-positive cells) in the co-cultured and IL-2-treated hIOs than in the control hIOs and that the mesenchyme of the co-cultured and IL-2-treated hIOs expressed higher levels of vascular endothelial growth factor (VEGF) (Supplementary Fig. 10d), which are known to recruit blood vessels from adjacent tissue^44^. Several studies have demonstrated that the AKT/mTOR and STAT3 pathways are related to the regulation of vascularization and vasculogenesis^45,46^. Therefore, downstream signaling events in hIOs influenced by the co-culture or IL-2-treatment, such as the AKT/mTOR and STAT3 pathways, may enable the hIOs to prepare for the fast intracellular signaling that is required to promote vascularization. However, this finding needs to be further investigated to determine whether and why the transdifferentiation of mesenchymal cells surrounding the hPSC-derived hIOs into vascular endothelial cells is dependent on in vivo transplantation, and this project is currently underway.

In summary, we report a simple method for forming mature hIOs from hPSCs in vitro. Our study highlights the importance of increasing the cellular complexity of existing organoid culture systems and the appropriate physiological functionality in order to better model adult human organ systems in vitro for facilitating the utilization of hIOs in various aspects of in vitro applications, such as a drug evaluation and pharmacological testing. Furthermore, this technology represents the first complete workflow linking hiPSC biobanks, and thus the opportunity for wide-scale genome editing, with the mature in vitro models of adult intestinal physiology, disease and potential clinical applications.

## Methods

### Cell culture and iPSC generation

Normal human fibroblasts (CRL-2097 and IMR90) and human T lymphocytes (Jurkat T cells) were obtained from the American Type Culture Collection. The H9 human embryonic stem cell (hESC) line was purchased from the WiCell Research Institute (Madison, WI, USA). Fibroblasts and hPSCs, including hESCs and hiPSCs, were cultured as described previously^47^. Integration-free hiPSCs were generated from fibroblasts using Episomal iPSC Reprogramming Vectors (Cat. No. A14703. Invitrogen, Carlsbad, CA, USA) as described previously^48^. Five days after electroporation, fibroblasts were seeded onto 6-well plates coated with Matrigel (BD Biosciences, San Diego, CA, USA) at a density of 1 × 10^5^/well in E8 medium (Stem Cell Technologies, Vancouver, Canada). After 3 weeks, hiPSC colonies were picked and expanded for further characterization. Jurkat T cells were cultured in RPMI 1640 medium (Invitrogen) containing 10% fetal bovine serum (FBS, Invitrogen), 1% penicillin-streptomycin (Invitrogen), and 2 mM L-glutamine (Invitrogen).

### Differentiation of hPSCs into hIOs

hIOs were generated as described previously^4,48^. To induce definitive endoderm identity, well-maintained hPSCs should be used and were treated with 100 ng/ml Activin A (R&D Systems, Minneapolis, MN, USA) for 3 days in RPMI 1640 medium with increasing concentrations of 0, 0.2, and 2% defined fetal bovine serum (dFBS, HyClone, Thermo Fisher Scientific Inc., Waltham, MA, USA). Cells were then treated for 4 days with RPMI 1640 medium containing 2% dFBS, 500 ng/ml FGF4 (R&D Systems), and 500 ng/ml WNT3A (R&D Systems) to promote differentiation into 3D hindgut spheroids. The spheroids were embedded in Matrigel (BD Biosciences) and cultured in hIO medium composed of advanced DMEM/F12 medium (Invitrogen) containing 1 × B27 (Invitrogen), 500 ng/ml R-Spondin 1 (R&D Systems), 100 ng/ml EGF (R&D Systems), and 100 ng/ml Noggin (R&D Systems), and then passaged every 2 weeks.

### Culture and in vitro maturation of hIOs

For the human T lymphocyte co-culture experiments, Jurkat T cells were stimulated with both 50 ng/ml phorbol myristate acetate (PMA; Sigma-Aldrich, St. Louis, MO, USA) and 500 ng/ml calcium ionophore A23187 (Sigma-Aldrich) for 3 h. A Transwell polyester membrane insert (pore size 0.4 μm, Corning, NY, USA) on which hIOs had been embedded within Matrigel (BD Biosciences) was placed into the well of a 12-well plate containing stimulated Jurkat T cells which had been seeded at 5 × 10^4^/cm^2^ in hIO medium. For achieving optimal efficiency and minimizing variations among cell lines, the effect of interleukin 2 (IL-2) on hIOs was assessed by using well-differentiated and characterized hIOs and adding freshly prepared rhIL-2 (R&D Systems) daily to hIO medium at a concentration of 1–8 ng/ml (approximately 13–104 U/ml) for 2 passages. To inhibit IL-2 signaling, hIOs were treated with 1 μg/ml of anti-IL-2Rγ_c_ or 3 μg/ml of anti-IL-2Rβ (R&D Systems). To block IL-2 downstream signal transduction, either the mTOR inhibitor Rapamycin (10 nM; Sigma-Aldrich) or one of the STAT3 inhibitors S3I-201 (10 μM; Sigma-Aldrich) or Stattic (1 μM; Sigma-Aldrich) were also added. To activate STAT3 signaling, rhIL-22 (1, 10 ng/ml, Peprotech, Rocky Hill, NJ, USA), and Colivelin (0.01, 1, 100 nM, Tocris Bioscience, Ellisville, MO, USA) was added in hIO culture medium for 2 passages. To estimate the size and number of budding structure of hIOs, we calculated the surface area using horizontal cross-sections of the organoids.

### Human phospho-kinase array

Protein phosphorylation was quantified using the Proteome Profiler Human Phospho-Kinase Array Kit (ARY003, R&D Systems) according to the manufacturer’s instructions. Protein extracts were prepared from untreated hIOs, hIOs in co-culture with human T lymphocytes and hIOs treated with 1 ng/ml rhIL-2. Briefly, hIOs were released from Matrigel using Cell Recovery Solution (Corning) and rinsed with ice cold PBS. hIOs were lysed at 4 °C for 30 min in Lysis Buffer of the Proteome Profiler Human Phospho-Kinase Array kit (R&D Systems). The phospho-kinase array membranes were blocked, incubated with 200 μg of total protein from hIOs overnight at 4 °C, and then incubated further with cocktails of biotinylated detection antibodies for 2 h at room temperature. Signal was detected with the ECL Plus Western Blotting Detection System (GE Healthcare, Buckinghamshire, UK) and the images obtained underwent quantification by densitometry with Image Gauge software (Fuji Photo Film GMBH, Tokyo, Japan) to determine phospho-protein levels.

### Measurement of cytokine secretion

Stimulated and unstimulated Jurkat T cells were cultured for 2 days. Culture medium from each was collected and levels of cytokines were determined using an enzyme-linked immunosorbent assay (ELISA) IL-2, IL-8, TNFα, IL-22, IL-6, IL-1β, IL-11, EGF, IL-10 (R&D Systems), and OSM (Abcam, CA, USA). ELISA was performed according to the manufacturer’s instructions and as described previously^49^ prior to quantification with a Spectra Max M3 microplate reader (Molecular Devices, Sunnyvale, CA, USA).

### Microarray

Microarray experiments were conducted according to the manufacturer’s protocol using the Low RNA input linear amplification kit, cRNA cleanup module and one-color platform (Cy3) Whole Human Genome Microarray 4 × 44 K (Agilent Technologies, Santa Clara, CA, USA) as described previously^49^. Gene expression data were processed using GeneSpringGX 7.3 (Agilent Technologies). The data were normalized using global scale normalization and differentially expressed genes were selected on the basis of a >2-fold change.

### RNA-sequencing (RNA-seq)

RNA samples were analyzed using an Agilent 2100 Bioanalyzer system (Agilent Biotechnologies). Only samples of high-quality RNA (RNA Integrity Number ≥ 7.5) were used in the following mRNA sample preparation for sequencing. Libraries were prepared using Illumina TruSeq library preparation per manufacturer specifications. Sample sequencing was performed on Illumina HiSeq2500 machines (Illumina, San Diego, CA, USA) using the standard Illumina RNA-Seq protocol with a read length of 2 × 100 bases. Sequencing quality was assessed with the FastQC package and then the reads containing adapters were trimmed using cutadapt and sickle for low-quality ends with a Phred quality threshold score of 20. If the trimmed read length was less than 50 bp, it was excluded. After filtering for sequencing errors, the processed reads were mapped to the reference genome using HISAT2 (v2.0.5) with default parameter settings. The human reference transcriptome annotation and reference genome from hg19 were used. Gene expression quantification was performed with Cuffquant and Cuffnorm (Cufflinks v2.2.1). Cuffdiff was used to analyze the differentially expressed genes (DEGs) between samples.

### Bioinformatic analysis

Hierarchical clustering and the heat map were generated using MeV v 4.9.0 software. Other bioinformatic analyses were performed using IPA analysis software (Ingenuity systems, Redwood City, CA, USA), the PANTHER (Protein ANalysis THrough Evolutionary Relationships, http://www.pantherdb.org/) database and DAVID Bioinformatics Resources 6.7 (http://david.abcc.ncifcrf.gov/). Differentially phosphorylated proteins were used for the analysis and visualization of functional interaction networks. Core pathways in the network were further analyzed using Reactome (Reactome FI software, http://apps.cytoscape.org/apps/reactomefis). The functionally grouped gene ontology (GO)/pathway was analyzed using the Cytoscape software platform (version 3.3.0, http://www.cytoscape.org/what_is_cytoscape.html) with the ClueGO plug-in (Version 2.2.5, http://apps.cytoscape.org/apps/cluego). Principle component analysis (PCA) was performed to visualize and quantify multi-dimensional variation between samples. Principle components were calculated using the function ‘prcomp’ found in the R (version 3.1.2) statistical programming language and plotted using the R package. Hierarchical clustering based on a Mcquitty linkage method and the Canberra distance was used to classify discrete samples according to the degree of total transcriptional dissimilarity. Spearman correlation was applied as an additional assessment of the cumulative degree of correlation among microarray or RNAseq datasets. We calculated the Spearman’s correlation in a pairwise variable for all samples. The Spearman correlation was plotted as a heatmap using the ‘heatmap.2’ function in the R package ‘gplots’.

### Quantitative RT-PCR (qPCR)

Total RNA was prepared using an RNeasy Kit (Qiagen, Valencia, CA, USA) and reverse-transcribed using a Superscript III cDNA synthesis kit (Invitrogen). qPCR was performed for triplicate samples using a 7500 Fast Real-time PCR system (Applied Biosystems). A house-keeping gene encoding glyceraldehyde-3-phosphate dehydrogenase (GAPDH) was used as an internal control. RNA extracted from adult human small intestine (hSI) (Clonetech, Fremont, CA, USA) was used as a positive control. The primers used in this study are listed in Supplementary Table 4.

### Western blotting

Cells were lysed with RIPA buffer and debris was removed by centrifugation at 4 °C before 20 μg of total protein was separated by electrophoresis on a 4–15% gradient gel (Ready Gel, Bio-Rad Laboratories, Hercules, CA) and transferred to a PVDF membrane. The antibodies used in this study are listed in Supplementary Table 5.

### Processing and immunofluorescence analysis of cells and hIOs

hPSCs and definitive endodermal cells were fixed in 4% paraformaldehyde (PFA) and then permeabilized with 0.1% Triton X-100 prior to immunofluorescence analysis. hIOs and tissues were fixed, cryo-protected in sucrose and frozen in optimal-cutting-temperature (OCT) compound (Sakura Finetek, Tokyo, Japan). Frozen sections were cut at a thickness of 10–20 μm using a cryostat microtome at −20 °C and permeabilized with 0.1% Triton X-100 for immunofluorescence analysis as described previously^50^. In brief, after being blocked with 4% BSA, samples were incubated with primary antibodies (Supplementary Table 5) at 4 °C overnight followed by incubation in the corresponding secondary antibodies for 1 h at room temperature. Paraffin sections were deparaffinized, subjected to antigen retrieval and stained in a similar fashion to OCT sections. Cell nuclei were visualized with DAPI (4′,6-diamidino-2-phenylindole). Slides were examined with a fluorescence microscope (IX51, Olympus, Japan), an Axiovert 200 M microscope (Carl Zeiss, Gottingen, Germany) and confocal microscope (Cat. No. FV1000 Live, OLYMPUS, Tokyo, Japan).

### Permeability assay

To determine permeability of hIOs, 4 kDa, and 40 kDa fluorescein isothiocyanate-dextran (FITC-dextran, Sigma Aldrich) was used. hIOs were washed with 5 times with PBS, and incubated with 1.25 μM FITC-Dextran for 30 min at 37 °C. FITC-dextran was removed, and hIOs were washed with PBS at least 5 times. A fluorescence microscope (IX51, Olympus) was used for imaging.

### P-glycoprotein (P-gp)/MDR1 activity assay

To determine P-glycoprotein transporter activity, at least 20 hIOs per group were used in triplicate. hIOs were placed into 4-well plates, washed three times with Hank’s balanced salt solution (HBSS with calcium and magnesium, pH = 7.4; Invitrogen) containing 25 mM HEPES and incubated at 37 °C for 30 min. The P-gp substrate paclitaxel, in DMSO (10 μM, Sigma-Aldrich), was added to hIO cultures and incubated for 2 h on a shaker at 50 r.p.m. in the presence or absence of verapamil, a P-gp inhibitor, in phosphate-buffered saline (PBS; 50 μM, Sigma-Aldrich). After incubation, hIOs were washed three times with HBSS and ruptured with an ultrasonic cell disruptor. The homogenate was centrifuged at 13,000×*g* for 10 min at 4 °C, and the resulting supernatant was collected for analysis. The concentration of paclitaxel in each sample was quantitated by an LC-ESI/MS/MS analysis using a 3200 QTRAP LC-MS/MS system (Applied Biosystems, Foster City, CA, USA) equipped with a Turbo V^TM^ Ion Spray source and an Agilent 1200 series HPLC system (Agilent Technologies).

### Calcium imaging with Fluo-4 AM

hIOs were loaded with Fluo-4 acetoxymethylester (fluo-4AM, 5 μM for 1 h, Molecular Probes, Eugene, Oregon, USA). hIOs were then washed with Ca^2+^-free isotonic buffer (140 mM NaCl, 5 mM KCl, 10 mM HEPES, 5.5 mM D-Glucose, 2 mM MgCl_2_) and placed on the stage of confocal microscope (FV1000 Live, Olympus). hIOs were stimulated with 50 mM glucose (Sigma Aldrich) in Ca^2+^-free isotonic buffer. hIOs were excited at 488 nm, and the signal emitted at 505–530 nm was recorded. The fluorescence intensity of the region of interest (ROI) was calculated using FV1000 software.

### Forskolin-induced swelling assay for CFTR function

To determine CFTR activity of hIOs, forskolin-induced swelling (FIS) assay was used as described previously^20^. hIOs were seeded in 4-well tissue culture plates with 10 μl Matrigel dome and 1 ml of culture medium. Two days after seeding, 25 μM of forskolin (Merck Millipore, Billerica, USA) was added in culture medium and hIOs were analyzed by live microscopy imaging (IX83, Olympus) to measure CFTR function. For CFTR inhibition, hIOs were pre-incubated with 100 μM CFTR Inhibitor-172 (Merck Millipore), and 100 μM CFTR Inhibitor II (GlyH-101, Merck Millipore) for 3 h. After pre-incubation, 25 μM forskolin was added and hIOs were directly analyzed.

### Mucicarmine and periodic acid-Schiff (PAS) staining

To detect mature goblet cells, we performed mucicarmine and PAS staining. Histological analyses were performed with 4% PFA fixed OCT-embedded organoid sections. Slides were subjected to mucicarmine (abcam) and PAS staining (Microscopy PAS staining kit, Merck Millipore) as recommended by the manufacturer.

### Hormone secretion assay for GIP

hIOs were washed with PBS 5 times, and hIO culture medium was added. After a 24-h incubation, the supernatant was harvested, and secreted GIP was measured using a human total GIP ELISA kit (Merck Millipore). DNA was extracted from hIOs and quantified using Nanodrop (Nanodrop2000c spectrophotometer, Thermo Fisher Scientific, Inc.). Total GIP content was normalized to DNA content. Genomic DNA was prepared using a DNeasy Kit (Qiagen).

### Transplantation

Eight to twelve-week-old NOD-SCID IL-2Rγnull (NSG) mice were used in all experiments (8–12 weeks old, Jackson Laboratories, Bar Harbor, ME, USA). All mice were housed in a standard animal maintenance facility at a constant temperature (20–22 °C) with a 12:12 h light: dark schedule. Ethical approval was received for all experiments from the Institutional Animal Care and Use Committee (IACUC) of KRIBB (Approval No: KRIBB-AEC-16206). Xenografting of hIOs under the kidney capsule was performed as described previously^10^. Briefly, hIOs were embedded into purified collagen type I (Rat tail collagen; BD Biosciences) for 12 h before transplantation. Mice were anesthetized with 2% isoflurane (Butler Schein, Dublin, OH, USA), and the left side of the mouse was then prepared using isopropyl alcohol and povidone-iodine in the standard fashion. A left subcostal incision was made to expose the kidney. The hIOs in the collagen plug were then transplanted into the subcapsular space of the kidney. The kidney was then returned to the peritoneal cavity and the mice were administered an IP flush with Enrofloxacin (5 mg/kg; Daehan New Pharm Co., Hwaseong-si, Korea). The skin was closed with a double layer and mice were kept warm with a heating pad until they had recovered fully from the anesthesia. Mice were euthanized humanely one week after transplantation and the xenografts were isolated for analysis.

### In vivo fluorescence imaging

To monitor the transplanted hIOs, hIOs were incubated with 1,1-dioctadecyl-3,3,3,3-tetramethylindotricarbocyanine iodide (DiR, Invitrogen) in 4-well plates at 37 °C for 15 min. After washing with PBS, hIOs were incubated with fresh medium and then transplanted under the kidney capsule. To visualize fluorescence in vivo, the recipient mice (*n* = 3) were anesthetized with 2% inhaled isoflurane (Terrell™, Piramal Healthcare, Bethlehem, PA, USA) one day after transplantation and placed into a light-sealed chamber connected to a charge-coupled device camera. To confirm the exact size of the transplanted hIOs, the kidneys of the recipient mice were isolated one week after transplantation and placed in a light-sealed chamber connected to a charge-coupled device camera. The fluorescence intensity of each region of interest was measured with the In Vivo Imaging System (IVIS Lumina II, Xenogen Corp., Alameda, CA, USA) with emission at 780 nm and excitation at 750 nm.

### In situ RNA hybridization and immunofluorescent staining

In situ RNA hybridization was conducted using RNAscope® Fluorescent Multiplex Assay (Advanced Cell Diagnostics, Hayward, CA) following the User Guide from the manufacturer. Briefly, 4% PFA–fixed 10-μm-thick frozen transplanted hIOs sections were mounted on SuperFrost® Plus slides (Thermo Fisher Scientific Inc.). The samples applied target retrieval with boiling for 5 min and were treated by Protease ΙΙΙ for 30 min at 40 °C. The slides were incubated with RNAscope® Hs-DEFA5 and Hs-OLFM4 probes (Supplementary Table 6) for 2 h at 40 °C, and the amplification followed the standard protocols. For simultaneous detection of mRNAs and proteins, immunofluorescence staining was performed on the same slide. The signal was visualized using an Olympus Spectroscopic Confocal Laser Scanning Microscope (FV1000 Live, Olympus).

### Measurement of episomal copy-number

hiPSC lysates were prepared using 1× Taq buffer (Takara, Kyoto, Japan) and proteinase K at 55 °C for 3 h and were used for qPCR analysis as described previously^51^. A known concentration of the pCXLE-hFbx15-cont2 plasmid was used to create a standard curve. The copy number of EBNA-1 and FBXO15 in each hiPSC line was calculated from the threshold cycle (Ct) values obtained over six replicates.

### Short tandem repeat (STR) and karyotype analysis

STR analysis was performed by HumanPass, Inc. (Seoul, Korea) using genomic DNA isolated from fibroblasts and the corresponding iPSC lines. G-banding karyotype analysis was performed by GenDix, Inc. (Seoul, Korea).

### In vivo differentiation via teratoma formation

A total of 1 × 10^6^ cells were mixed with Matrigel and injected subcutaneously into the dorso-lateral area of BALB/c nude mice (6 weeks old, Orient Bio, Inc., Seongnam, Korea). After 8 to 10 weeks, the resulting teratomas were dissected, fixed in 4% PFA and embedded in paraffin. Paraffin-embedded teratomas were sectioned and then stained with hematoxylin and eosin solution (Sigma-Aldrich). Animal experiments were approved by the IACUC of KRIBB (Approval No: KRIBB-AEC-15192).

### Isolation and culture of NSG murine intestinal organoids

Pieces of small intestine (~0.5 mm) from NOD-SCID IL2 receptor γ chain^null^ (NSG) mice (The Jackson Laboratory, Bar Harbor, ME, USA) were incubated for 20 min at room temperature in Gentle Cell Dissociation Reagent (StemCells Inc., Newark, CA, USA). Isolated crypts were obtained through a 70-μm strainer and seeded into mixed solution with 1:1 growth factor-reduced (GFR) Matrigel (BD Biosciences) and IntestiCult™ Organoid Growth Medium (StemCells Inc.) in 24-well Clear TC-Treated Multiple Well Plates (Costar, Washington, DC, USA). The medium was changed every 3–4 days, and mIOs were passaged every 1 week. To evaluate the effects of murine IL-2 (mIL-2) on mIOs, organoids were treated with recombinant mIL-2 (1–20 ng/ml, Peprotech). Organoid morphology was observed using microscopy (DMI 4000B, Leica, Wetzlar, Germany).

### Isolation and analysis of lamina propria lymphocytes

Isolation of mouse lamina propria experiments were performed after approval by the Institutional Animal Care and Use Committee (IACUC) of KRIBB (approval No: KRIBB-AEC-17195), and adult C57BL/6 J mice (10 weeks old, Dae Han Bio Link Co., Ltd., Eumseong-gun, Chungcheongbuk-do, Korea) were used. Mouse small intestine lamina propria lymphocytes were isolated from five independent mice per experiment as described previously^52^. Isolated lamina propria lymphocytes were incubated in protein transport inhibitor (GolgiStop, BD Biosciences) for 6 h, then washed twice with staining buffer and blocked with Fc Blocking solution (BD Biosciences). The cells were labeled with surface antibodies at 4 °C for 30 min (Supplementary Table 5) and washed. For IL-2 staining, cells were subject to 20 min fixation at 4 °C using the fixation/permeabilization solution (BD Biosciences), washed twice with staining buffer and incubated with anti-mIL-2 antibody diluted in BD Perm/Wash (BD Biosciences) buffer for 1 h at 4 °C. Then, lamina propria lymphocytes were analyzed on a FACSCalibur (BD Biosciences) according to the manufacturer’s instructions. The data were analyzed with FlowJo V10 software (FLOWJO, Ashland, OR, USA).

### Statistical analysis

All results are expressed as mean ± standard error of the mean (SEM), and all experiments were repeated at least three times. *P* values were determined using two-tailed *t*-tests. All analyses of statistical significance were calculated and compared with the control group unless otherwise stated.

### Data availability

The data supporting the findings presented in this study are included in the manuscript and supplementary files. All microarray data are deposited in GEO under accession number GSE116738. Other relevant source data are available from the authors on request.

## Electronic supplementary material

Supplementary Information
