# Supplementary material for: Interleukin-2 induces the in vitro maturation of human pluripotent stem cell-derived intestinal organoids
Source: Nat Commun. 2018 Aug 2;9:3039. doi: 10.1038/s41467-018-05450-8 (PMC6072745; doi:10.1038/s41467-018-05450-8)
Supplement: Supplementary file 1 — Supplementary Information [file 41467_2018_5450_MOESM1_ESM.pdf]

Supplementary Information

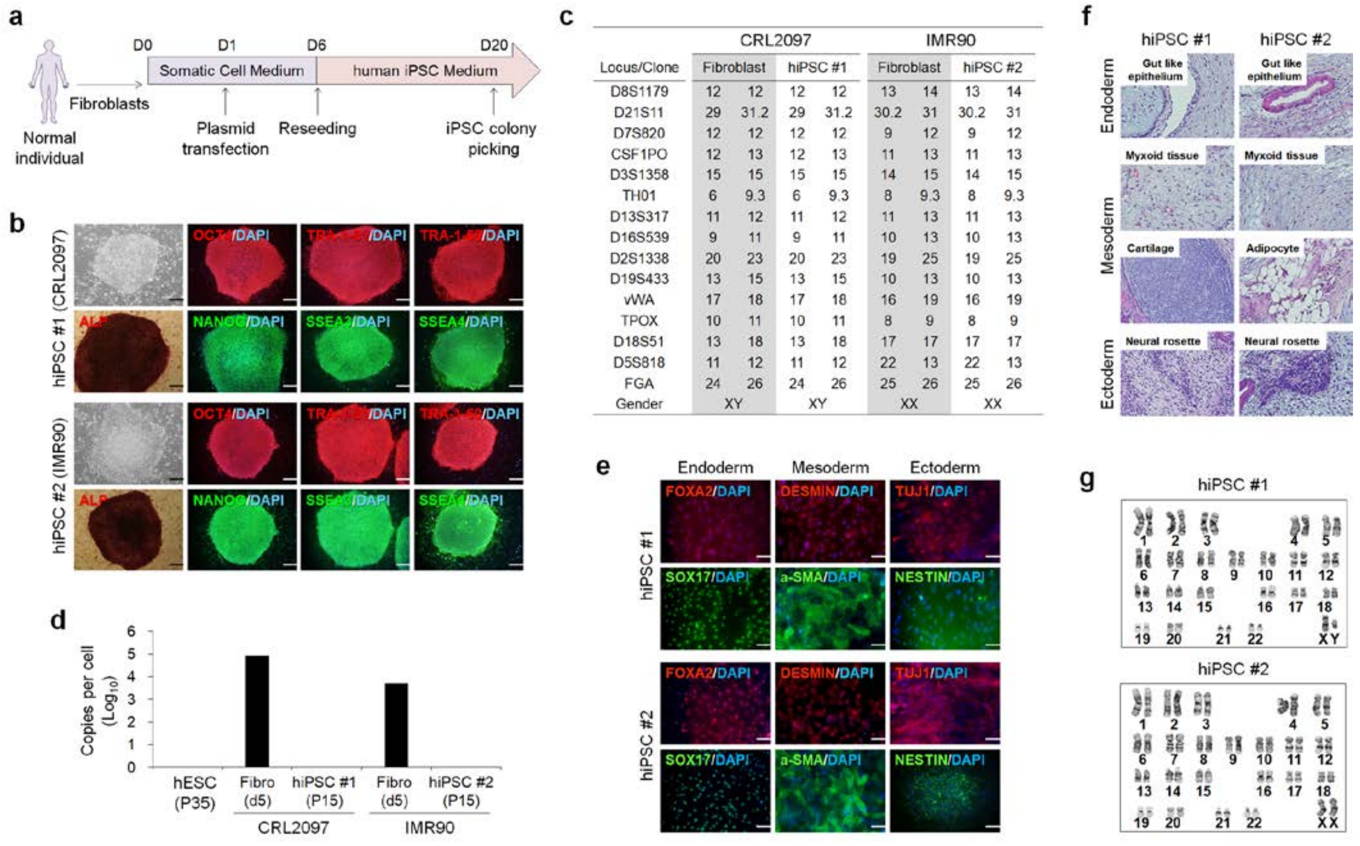

	CRL2097				IMR90			
Locus/Clone	Fibroblast		hiPSC #1		Fibroblast		hiPSC #2	
D8S1179	12	12	12	12	13	14	13	14
D21S11	29	31.2	29	31.2	30.2	31	30.2	31
D7S820	12	12	12	12	9	12	9	12
CSF1PO	12	13	12	13	11	13	11	13
D3S1358	15	15	15	15	14	15	14	15
TH01	6	9.3	6	9.3	8	9.3	8	9.3
D13S317	11	12	11	12	11	13	11	13
D16S539	9	11	9	11	10	13	10	13
D2S1338	20	23	20	23	19	25	19	25
D19S433	13	15	13	15	10	13	10	13
vWA	17	18	17	18	16	19	16	19
TPOX	10	11	10	11	8	9	8	9
D18S51	13	18	13	18	17	17	17	17
D5S818	11	12	11	12	22	13	22	13
FGA	24	26	24	26	25	26	25	26
Gender	XY		XY		XX		XX	

Supplementary Figure 1. Generation and characterization of hiPSC lines. (**a**) Schematic diagram of the reprogramming protocol. Human fibroblasts were reprogrammed into iPSCs using non-integrating episomal vectors. (**b**) Representative morphology and immunofluorescence analysis of hiPSC #1 derived from CRL2097 and hiPSC #2 derived from IMR90 for the pluripotency markers OCT4, NANOG, TRA-1-60, TRA-1-81, SSEA-3 and SSEA-4. All hiPSC colonies expressed alkaline phosphatase (ALP). Scale bar, 100 μm. (**c**) STR profiles of the hiPSC lines. (**d**) Copy number analysis of episomal vectors in hiPSC lines. The passage number is shown in parentheses. Fibroblasts were analyzed 5 days after electroporation as positive controls. (**e**) *In vitro* differentiation of hiPSC lines. Immunofluorescence analysis of the endodermal markers FOXA2 and SOX17, the mesodermal markers DESMIN and α-smooth muscle actin (α-SMA), and the ectodermal markers TUJ1 and NESTIN in hiPSC lines. Nuclei were stained with DAPI (blue). Scale bar, 100 μm. (**f**) *In vivo* differentiation *via* teratoma formation. Histological analysis of teratomas derived from hiPSCs by hematoxylin and eosin staining. (**g**) Karyotype analysis of hiPSC lines.

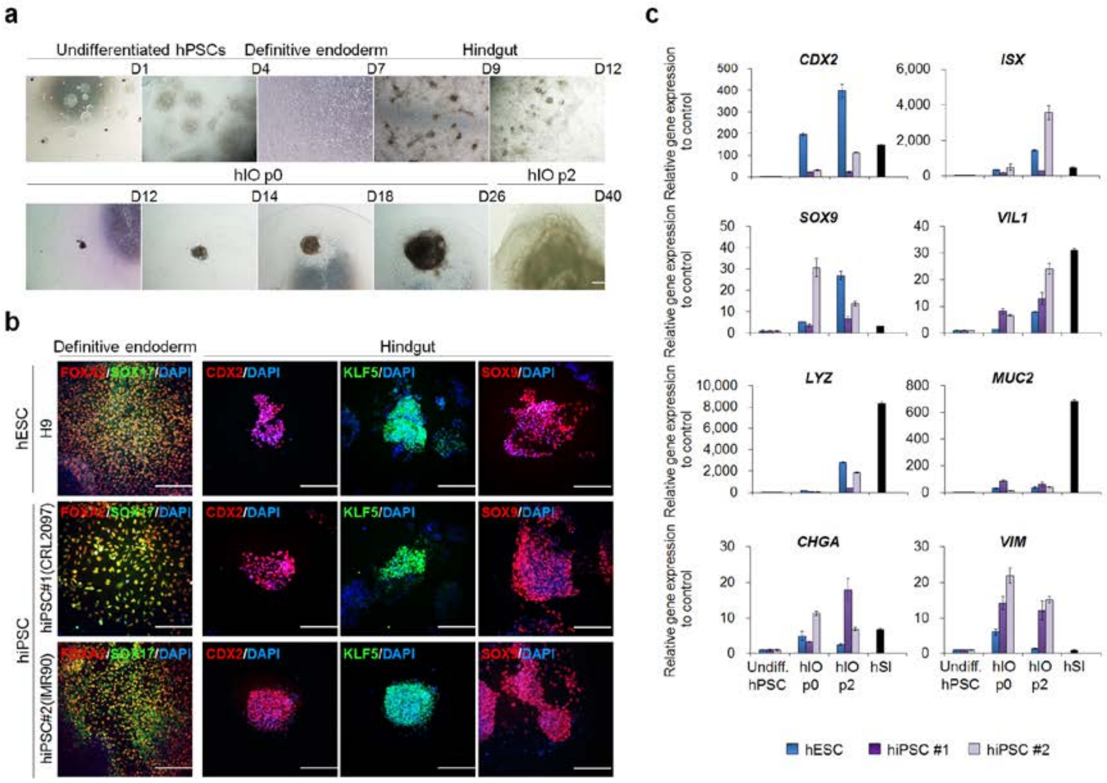

Supplementary Figure 2. Efficient directed differentiation of hPSCs into hIOs. (**a**) Representative images of the directed differentiation of hPSCs into definitive endoderm (DE), hindgut (HG) and hIOs. Scale bar, 200 μm. (**b**) Immunofluorescent staining of DE (FOXA2, SOX17) and HG markers (CDX2, KLF5, SOX9) during directed differentiation. Scale bar, 200 μm. (**c**) qPCR analysis of intestinal markers in undifferentiated hPSCs (three independent hPSC lines: H9 hESC, hiPSC #1 derived from CRL2097 and hiPSC #2 derived from IMR90), hPSC-derived differentiated hIO (p0, p2), and hSI. Data are presented as mean values of replicates ± SEM.

a

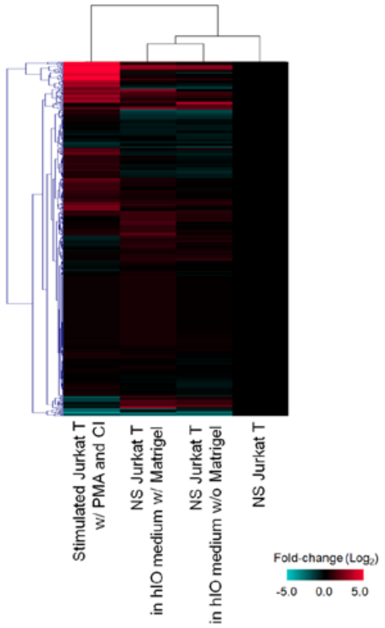

b

Gene Symbol	NS Jurkat T	Stimulated Jurkat T w/ PMA and CI	NS Jurkat T in hIO medium w/ Matrigel	NS Jurkat T in hIO medium w/o Matrigel
IL2	1.00	247.30	1.38	1.08
IL8	1.00	169.47	1.38	1.08
TNF	1.00	147.13	4.24	3.37
IL22	1.00	30.99	1.38	1.08
IL6	1.00	14.73	1.38	1.08
IL1B	1.00	5.42	5.11	1.08
IL11	1.00	4.57	2.47	1.47
EGF	1.00	1.18	1.38	1.08
OSM	1.00	1.09	1.16	1.59
IL10	1.00	0.84	1.02	1.06

Supplementary Figure 3. Gene expression profile of Jurkat T cells in various conditions. (**a**) Heatmaps of 759 genes encoding cytokines and chemokines in non-stimulated (NS) Jurkat T cells, PMA/ionophore stimulated Jurkat T cells, Jurkat T cells in hIO medium with Matrigel, and Jurkat T cells in hIO medium without Matrigel. (**b**) Gene expression of cytokines including *IL-2, IL-8, TNFα, IL-22, IL-6, IL-1β, IL-11, EGF, OSM,* and *IL-10* as assessed by microarray.

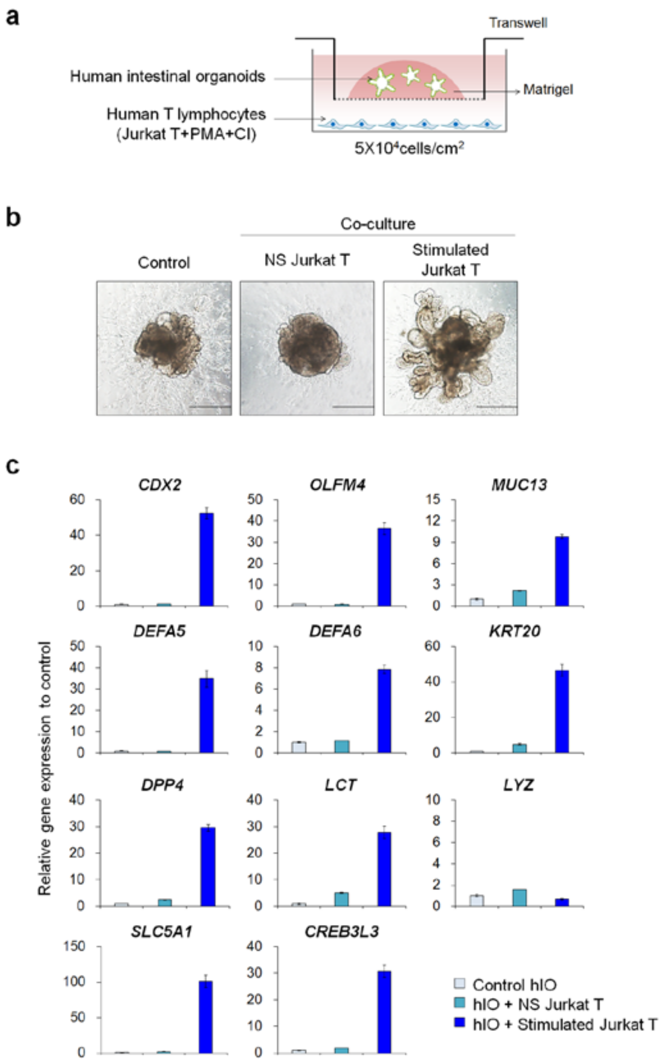

Supplementary Figure 4. *In vitro* maturation of hIOs by co-culture with stimulated Jurkat T cells. (**a**) Schematic of the co-culture system for hIOs and Human T lymphocytes. Matrigel-embedded hIOs placed on culture inserts were applied to wells containing PMA/ionophore-stimulated Jurkat T cells. (**b**) Representative images of the morphology of control hIOs, hIOs co-cultured with non-stimulated (NS) Jurkat T cells and stimulated Jurkat T cells. Scale bar, 500 μm. (**c**) qPCR analysis of the gene expression for intestinal maturation markers in control, co-cultured with NS Jurkat T cells or stimulated Jurkat T cells. Data are presented as mean values of replicates ± SEM.

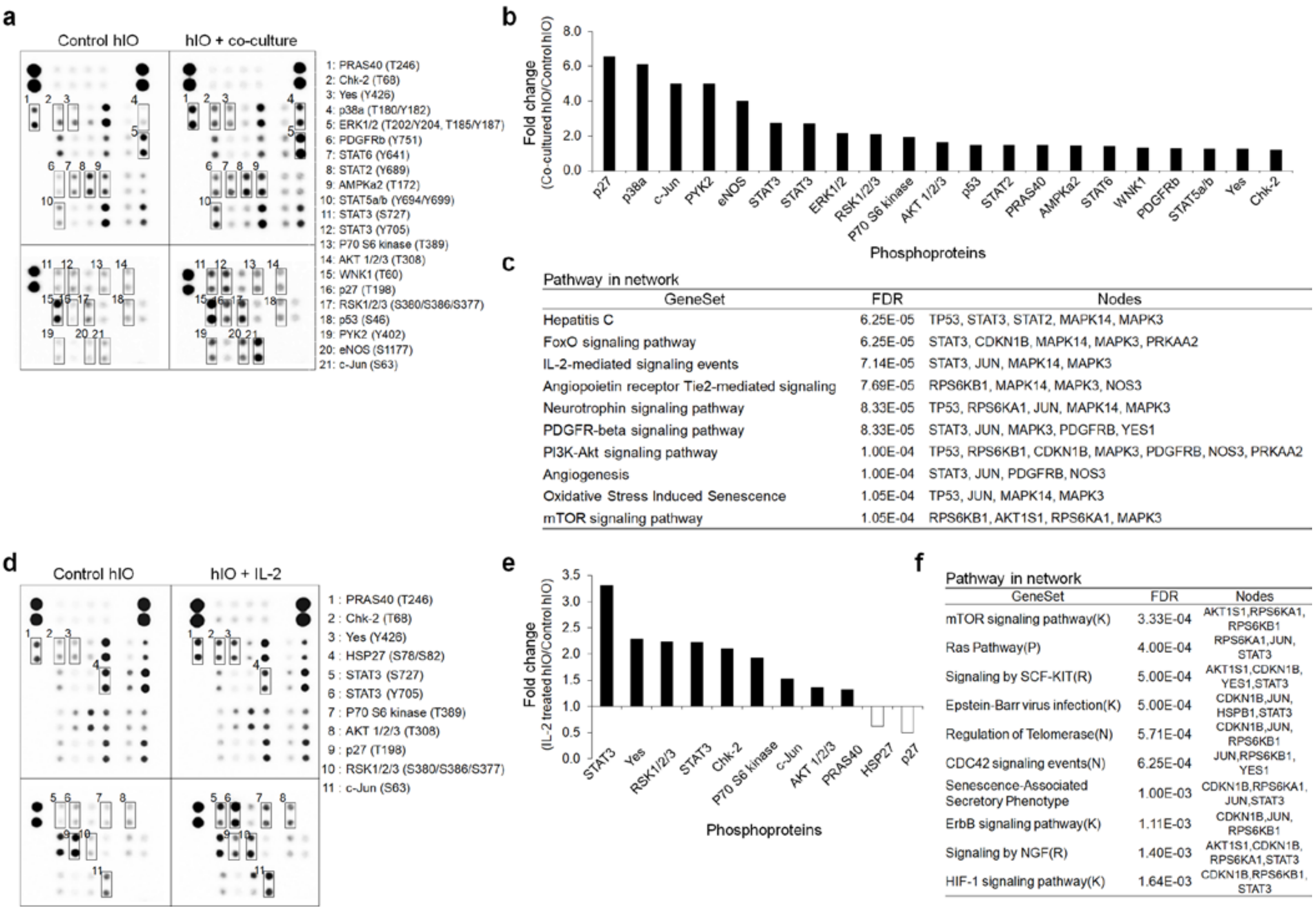

Supplementary Figure 5. Human phospho-kinase array to detect proteins phosphorylated in hIOs following co-culture with immune cells (a-c) or IL-2 treatment (d-f). (**a** and **d**) Human phospho-kinase array membranes. Each antibody was assessed in duplicate. (**b** and **e**) Graph showing fold change of densitometric values with respect to control hIOs. (**c** and **f**) Pathway enrichment analysis for differentially phosphorylated proteins in the co-cultured hIOs with false discovery rate (FDR) <0.001 and the IL-2-treated hIOs with FDR <0.05.

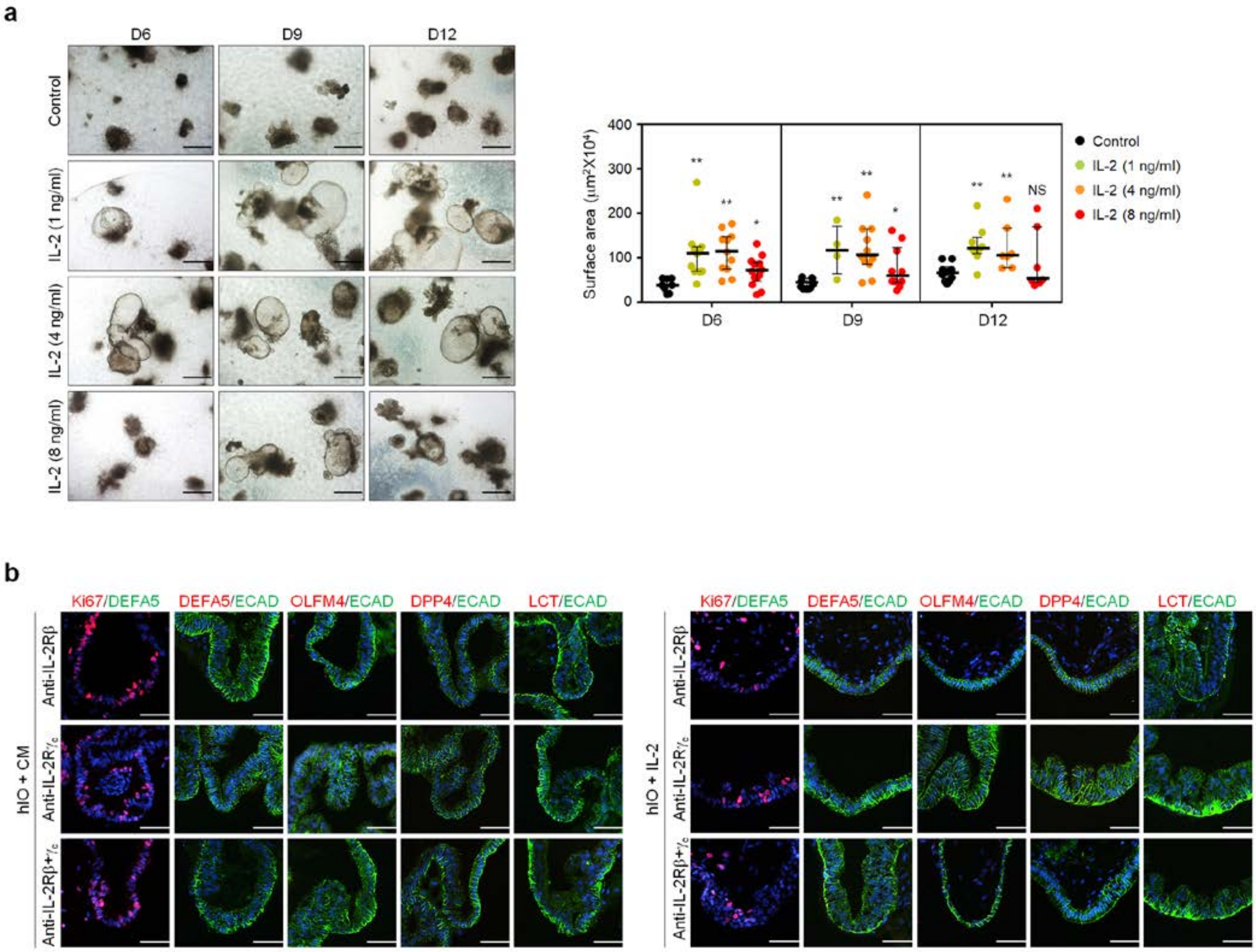

Supplementary Figure 6. Effect of IL-2 on the growth of hIOs. (**a**) Response of hIOs to varying concentrations of IL-2. Representative images of the morphology of hIOs after treatment with the indicated concentrations of rhIL-2 (1, 4 and 8 ng/ml; left panels). Quantitative assessment of the size of hIOs (6, 9, and 12 days at p2); n = 10 hIOs per group (right panels). (**b**) Immunofluorescent staining for the proliferation marker (Ki-67), intestinal maturation markers (DEFA5, OLFM4, DPP4 and LCT) in hIOs cultured with conditioned medium (CM) of stimulated Jurkat T cells or IL-2 treatment with IL-2 receptor inactivating antibodies (anti-IL-2Rβ, anti-IL-2Rγc). Scale bar, 50 μm.

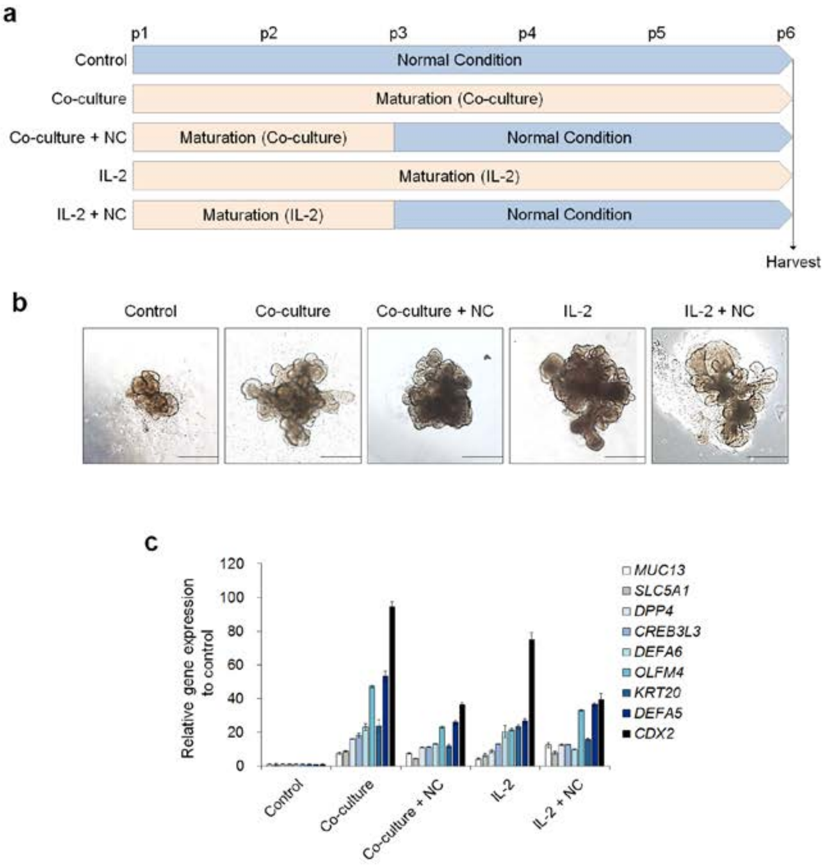

Supplementary Figure 7. The maturation status of hIOs was maintained after two passages under *in vitro* maturation condition. (**a**) Schematic diagrams for determining the maturation status of hIOs. (**b**) Representative morphologies under each condition. Scale bar, 500 μm. (**c**) qPCR analysis of the gene expression for intestinal maturation markers in hIOs under normal condition (NC) and maturation condition during four passages after two passages under *in vitro* maturation condition. Fold changes in expression level are relative to control hIOs. Data are presented as mean values of replicates ± SEM.

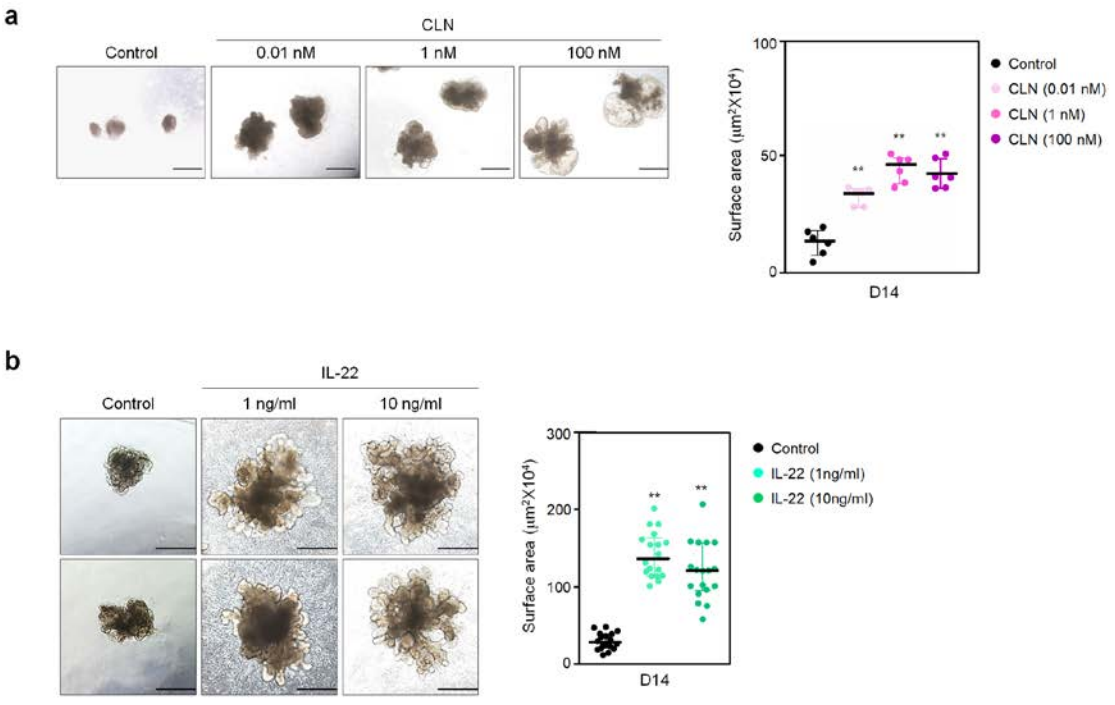

Supplementary Figure 8. Effect of STAT3 signaling on the growth of hIOs. (**a**) Response of hIOs to varying concentrations of Colivelin (CLN). Representative images of the morphologies of hIOs after treatment with the indicated concentrations of CLN (0.01, 1 and 100 nM; left panels). Quantitative assessment of the size of hIOs (14 days at p3); n = 6 hIOs per group (right panels). (**b**) Representative images of morphologies of hIOs after treatment of IL-22 (1 and 10 ng/ml; left panels). Quantitative assessment of the size of hIOs (14 days at p1); n = 18 hIOs per group (right panels). Scale bar, 1 mm. Data are presented as mean values of replicates ± SEM. $^{**}p < 0.01$ and $^{*}p < 0.05$ according to t-test.

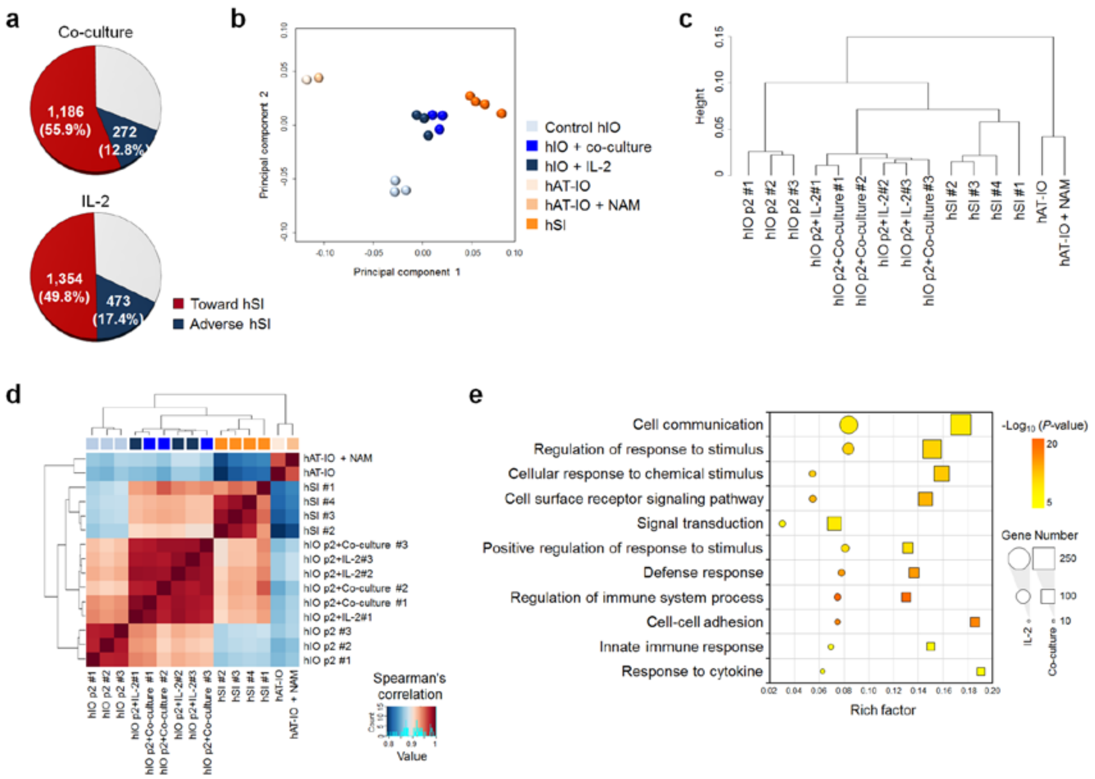

Supplementary Figure 9. Microarray analysis for assessment of the *in vitro* maturation of hIOs. (**a**) Quantitative representation of gene expression changes (two-fold cut-off) in hIOs following co-culture (n = 2) or treatment with IL-2 (n = 3) compared to control hIOs. Red represents an increase in expression, indicating increased similarity to hSI, blue represents a decrease in expression, indicating decreased similarity to hSI, and white denotes no difference in expression to control hIOs. Note that the majority of gene expression changes were increases (red), indicating increased similarity to the gene expression profile of hSI. A small subset of genes in each case displayed decreased expression. (**b**) PCA of microarray datasets for control hIOs (n = 3), co-cultured hIOs (hIO + co-culture, n = 3), IL-2 treated hIOs (hIO + IL-2, n = 3), adult human small intestine (hSI) (n = 4) and human adult tissue-derived intestinal organoids (hAT-IOs, n = 2) cultured in the presence or absence of nicotinamide (NAM). NAM was used to improve the culture efficiency of hAT-IOs. (**c**) A dendrogram based on hierarchical clustering of the gene sets from microarray using Canberra distance. Branch lengths indicate the degree of difference between samples. (**d**) Spearman correlation was used to cluster samples and generate a heatmap. Red indicates the highest level of similarity between samples and blue indicates the lowest level of similarity. (**e**) Top enriched Gene Ontology (GO) terms of genes associated with co-culture or treatment of IL-2. The size of the squares and circles presented

the number of selected genes of co-culture and IL-2, respectively. The color tints indicated the *P*-values of GO terms. Rich factor expressed the percentage of DEGs in co-culture or IL-2-associated genes, respectively, among the total number of genes in each GO term.

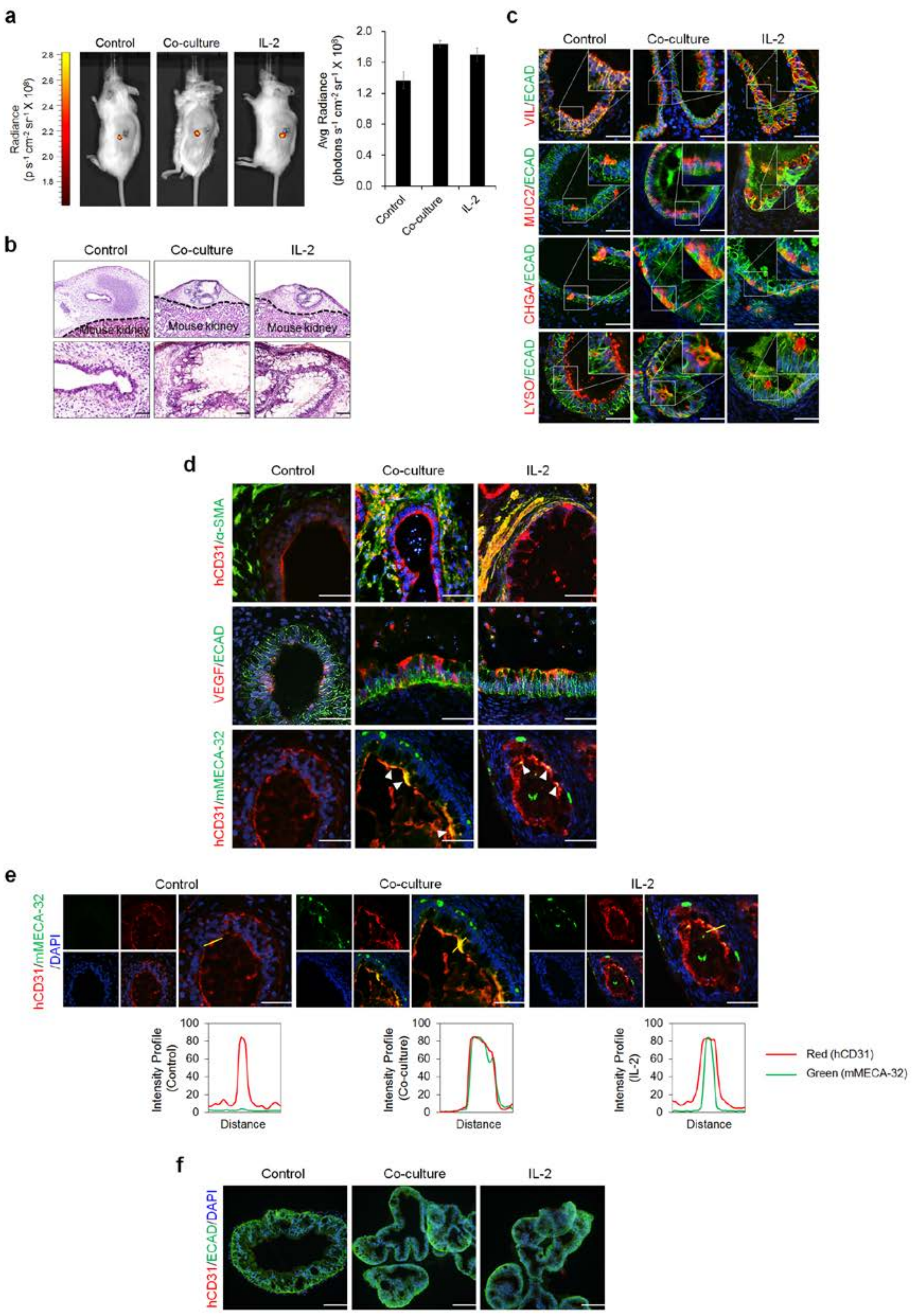

Supplementary Figure 10. Transplantation of *in vitro*-matured hIOs. (**a**) *In vivo* fluorescence analysis at 1 day post-transplantation of DiR-labeled hIOs into the kidney capsule of immunodeficient NSG mice (upper panels). Average radiance of mice transplanted with DiR-labeled hIOs (n = 3; lower panels). (**b**) Hematoxylin and eosin staining of hIOs 1 week after transplantation into the kidney capsule. (**c**)

Immunofluorescent staining for markers of enterocytes (VIL), goblet cells (MUC2), enteroendocrine cells (CHGA) and Paneth cells (LYSO). All four intestinal lineages were present in the transplanted hIOs. Immunofluorescent staining with E-cadherin (ECAD) was used to identify transplanted cells. Scale bar, 50 μm. (**d**) Immunofluorescent staining of the engrafted hIOs for markers of vasculature. Co-staining for human vascular endothelial cells (hCD31) and laminated smooth muscle (α-SMA; upper panels) around control, co-cultured and IL-2-treated hIOs 1 week after transplantation. Immunofluorescent staining for VEGF (middle panels) in control, co-cultured and IL-2-treated hIOs following transplantation. Immunofluorescent staining of blood vessels with hCD31 (human cells) and mMECA-32 (mouse vasculature) to determine connections between the *in vitro*-matured hIOs and host vasculature control, co-cultured and IL-2-treated hIOs 1 week after transplantation (bottom panels, white arrowheads). Cell nuclei (blue) were stained with DAPI. Scale bar, 50 μm. Similar results were obtained in three independent experiments, and representative images are shown. (**e**) Representative intensity profiles of hCD31 and mMECA-32 immunofluorescent staining; yellow line in the merged image denotes the axis of analysis. (**f**) Immunofluorescent staining of human-specific pan-endothelial CD31 (hCD31) to identify human vascular endothelial cells in hIOs prior to *in vivo* transplantation. Scale bar, 100 μm.

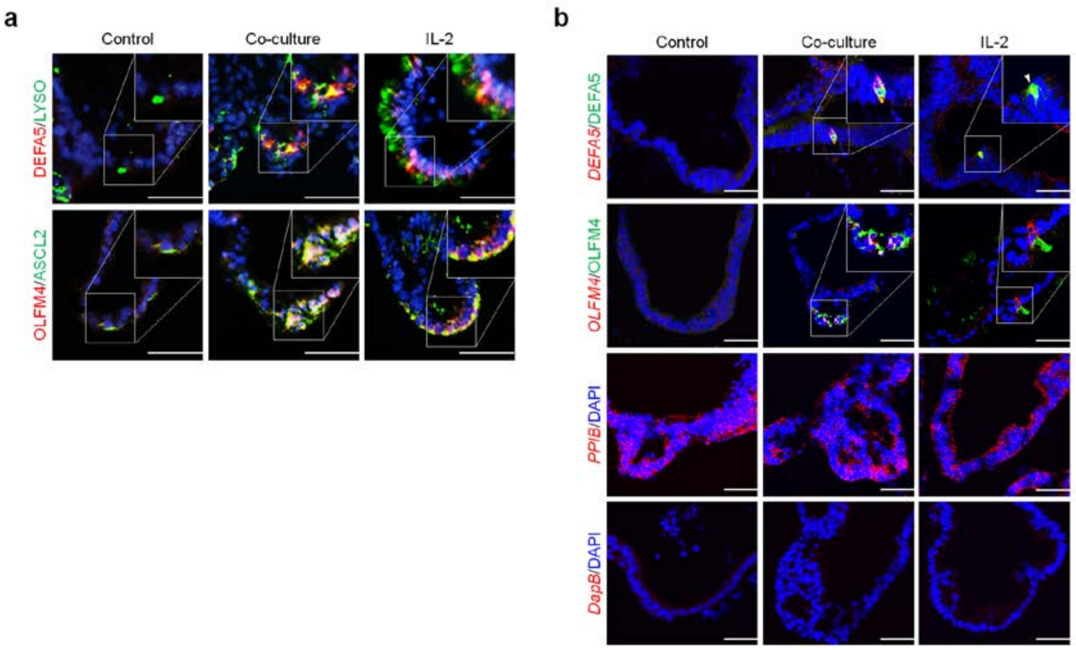

Supplementary Figure 11. Specific expression of DEFA5 and OLFM4 in co-cultured and IL-2 treated hIOs. (**a**) Co-staining of DEFA5 with lysozyme (LYZ, a Paneth cell marker) and OLFM4 with ASCL2 (an intestinal stem cell marker). (**b**) Dual RNAscope fluorescence in situ hybridization (FISH) and immunofluorescent staining for simultaneous detection of mRNAs (red signal) and proteins (green signal) for DEFA5 and OLFM4. Positive control stained for *PPIB* (a house keeping gene) and negative control stained for a bacterial *DapB* in hIOs. Cell nuclei (blue) were stained with DAPI. Scale bar, 50 μm.

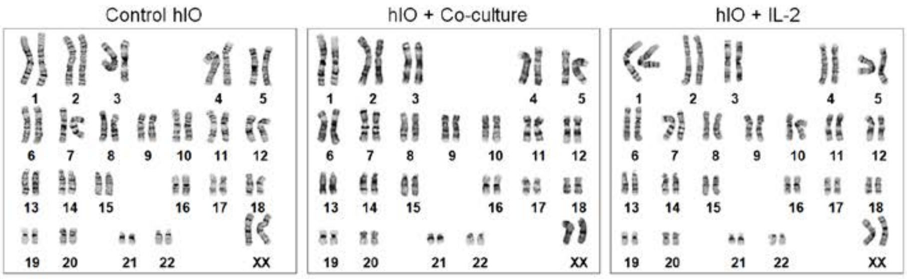

Supplementary Figure 12. Karyotype analysis of *in vitro*-matured hIOs.

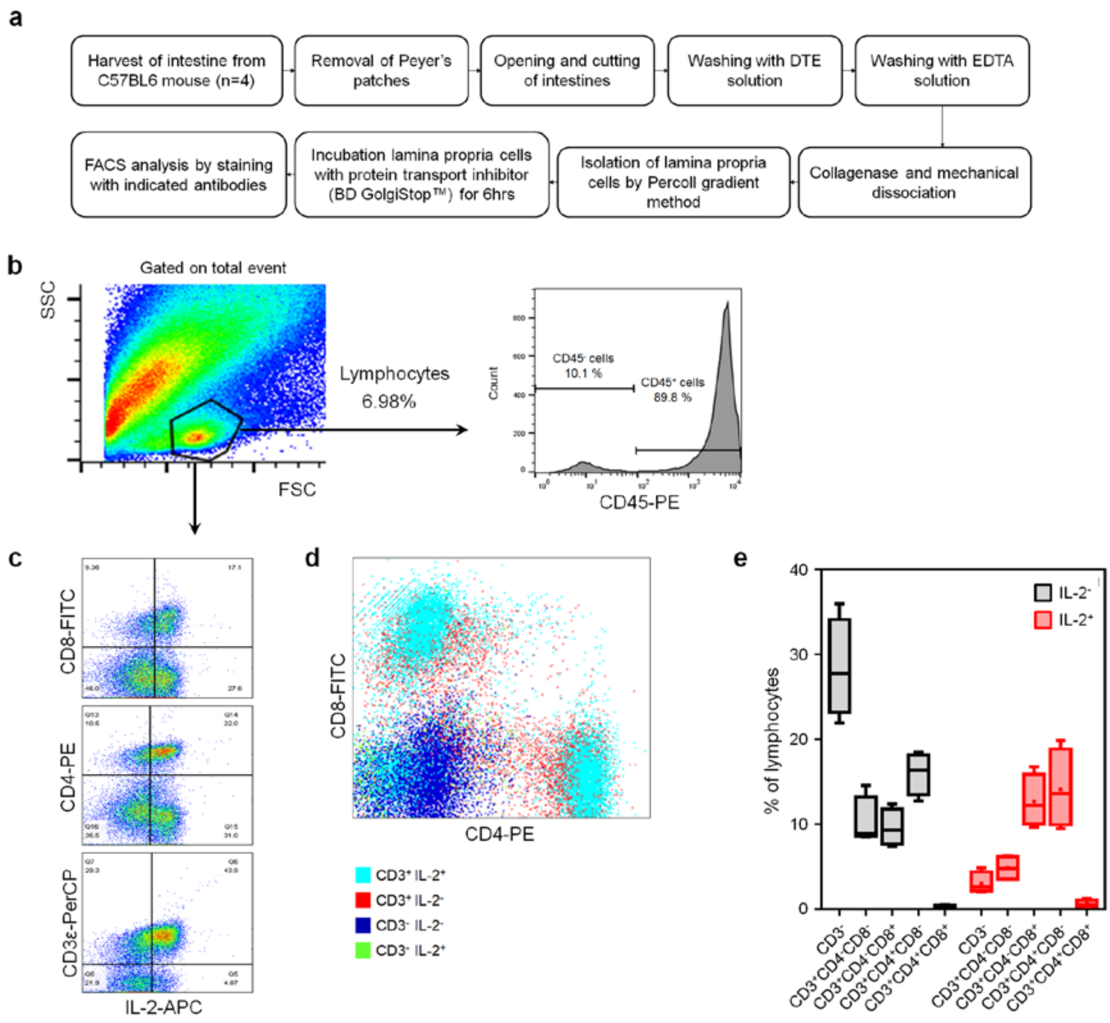

Supplementary Figure 13. Flow cytometry analysis of IL-2 secreting cells in mouse intestinal lamina propria lymphocytes. (**a**) Flow chart depicts the isolation of intestinal lamina propria lymphocytes and analysis of IL-2 secreting cells. (**b**) Forward and side scatter properties for gating lymphocytes based on total events. Histogram graph of FACS analysis of $CD45^+$ cells in the gated lymphocytes. (**c**) Pseudo-color scatter plot of staining cells using mAbs that are specific for CD3ε, CD4, CD8 and IL-2. (**d**) Cell subpopulations secreting IL-2 were distinguished on the basis of different immune cell surface markers, revealing $CD4^+$ T cells and $CD8^+$ T cells among $CD3^+$ T cells. (**e**) A graph for the proportion of cell subpopulation of intestinal lamina proporia lymphocytes based on IL-2 secretion.

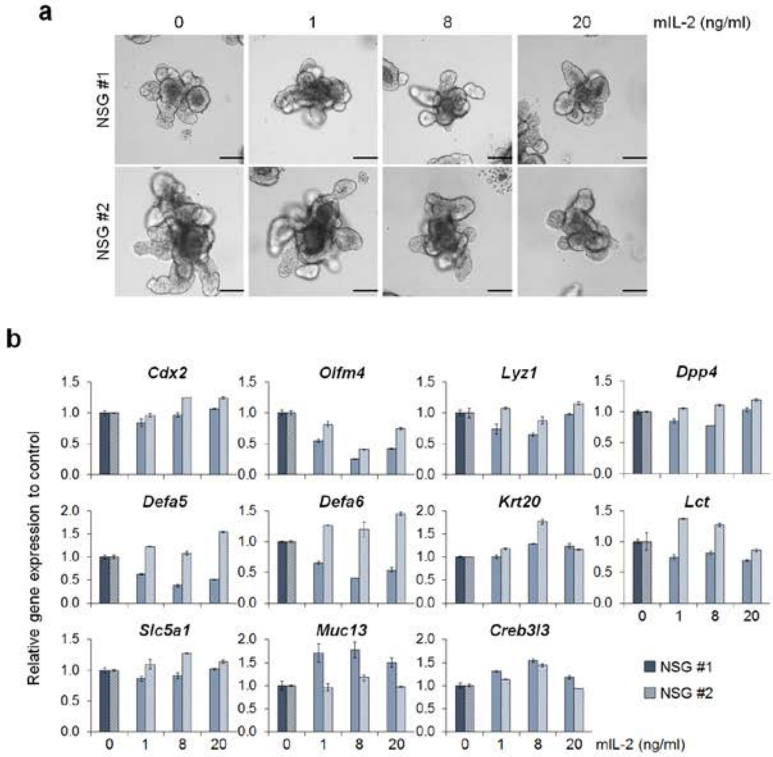

Supplementary Figure 14. IL-2 receptor specificity for intestinal maturation using the murine intestinal organoids. (**a**) Representative images of morphology of murine intestinal organoids (mIOs) derived from immunodeficient NSG (NOD/SCID IL-2 receptor gamma chain knockout) mice with mIL-2 (1, 8, 20 ng/ml). Scale bar, 100 μm. (**b**) qPCR analysis of the expression of intestinal and maturation markers in mIL-2-treated mIOs. Data are presented as mean values of replicates ± SEM.

Supplementary Table 1. The expression level of genes known to be involved in the defense response, digestive function and intestinal markers.

Defense response

Gene Symbol	DE	HG	hIO p0	hIO p2	Co-culture	IL-2	hAT-IO	hAT-IO+ NAM	hSI	Agilent Probe ID
ANG	0.31	0.17	1.68	9.80	8.97	11.39	4.37	3.39	3.64	A_33_P3236177
CD48	52.89	3.21	0.71	0.83	0.60	1.28	0.63	0.64	72.27	A_32_P175934
CD74	0.10	0.09	0.25	1.01	0.56	0.56	1.57	0.64	21.62	A_23_P70095
CD84	0.87	0.62	0.96	1.13	0.81	0.88	0.90	0.92	8.60	A_23_P361940
CLEC1A	0.32	0.17	6.03	5.22	0.16	3.23	0.17	0.18	15.22	A_33_P3295333
CST3	1.30	1.31	1.50	5.68	12.51	6.89	4.26	3.91	8.70	A_33_P3228266
CX3CL1	0.42	0.71	1.47	3.42	1.82	4.39	0.84	0.71	4.51	A_24_P390495
CYSLTR1	1.06	0.87	1.65	4.44	2.85	2.63	0.74	0.28	8.57	A_23_P22660
DEFA5	2.36	0.62	0.96	1.13	243.51	6.58	0.73	1.39	91467.19	A_23_P112086
DEFA6	0.87	9.00	0.96	1.13	207.47	13.47	0.73	0.73	93678.16	A_24_P363711
DEFB1	0.47	0.48	1.76	3.12	24.77	13.13	3.28	1.18	15.80	A_23_P71480
HLA-B	0.42	0.50	0.37	0.72	2.07	0.64	1.94	1.77	5.03	A_33_P3424800
ICOSLG	3.08	0.68	0.49	1.10	2.06	1.41	3.10	2.49	1.55	A_23_P317667
IL32	0.39	0.14	0.46	1.19	16.48	4.17	9.19	9.85	14.78	A_23_P15146
INHBA	0.73	2.08	2.19	1.50	2.82	0.64	0.01	0.13	0.30	A_23_P122924
INHBB	0.30	1.48	15.76	2.71	2.69	6.31	0.40	1.64	5.61	A_23_P153964
KCNN4	0.12	0.22	0.39	9.65	13.84	8.41	42.44	35.38	3.73	A_23_P67529
KREMEN1	0.51	0.86	3.76	2.09	3.51	4.21	2.16	2.54	4.75	A_23_P68851
LILRA2	0.87	0.75	0.96	1.13	0.81	0.88	0.91	0.93	6.15	A_23_P142205
LILRB3	0.72	0.43	1.81	0.66	0.17	0.60	0.12	0.05	12.33	A_32_P70158
LILRB5	0.73	0.52	6.46	0.95	0.69	0.74	0.63	0.63	841.45	A_23_P4773
LSP1	0.27	0.35	17.44	18.05	3.05	4.48	3.43	4.33	8.94	A_33_P3369567
MST1R	0.13	0.12	0.20	2.29	15.13	4.05	14.89	15.58	4.38	A_23_P256312
MX2	0.20	0.34	0.10	0.23	0.71	0.54	0.61	0.61	2.19	A_33_P3278200
NLRP1	1.12	2.01	0.74	0.99	1.07	0.67	0.25	0.17	7.96	A_23_P89550
NLRP3	0.66	0.42	3.28	0.52	1.07	0.49	0.41	0.50	13.83	A_33_P3281695
NOD1	0.29	0.55	0.71	0.52	0.64	0.47	0.40	0.16	1.54	A_24_P129277
NOD2	0.36	0.26	0.91	1.10	1.34	1.72	0.14	0.11	7.38	A_23_P420863
REG3A	1.16	1.06	0.68	7.49	45.07	0.67	0.25	0.26	26132.02	A_23_P119936
RELA	1.31	0.99	2.26	3.77	2.29	2.42	1.66	1.85	1.60	A_33_P3209433
RNASE6	0.26	0.19	1.42	4.59	59.61	13.07	14.51	4.78	54.28	A_23_P3014
SP140	3.77	0.20	1.14	4.69	6.20	3.11	4.06	4.16	13.76	A_24_P328504
TAP1	0.07	0.06	0.16	0.23	0.97	0.31	0.89	0.85	1.77	A_23_P59005
TAPBP	0.26	0.24	0.59	1.69	3.52	2.50	1.40	1.14	3.08	A_23_P259580
TFF3	0.21	1.19	14.39	1339.10	2120.16	1901.30	1742.15	1440.17	378.19	A_33_P3334305
TLR1	1.02	0.62	1.91	4.61	7.45	4.27	2.88	0.55	39.06	A_23_P10873
TLR3	0.12	0.13	0.20	0.80	14.46	2.61	15.56	9.38	6.72	A_23_P29922
TNF	0.28	0.84	3.02	2.97	4.04	1.13	11.25	12.87	7.12	A_23_P376488
TNFRSF1A	1.03	0.77	0.67	1.30	2.47	1.30	0.84	0.88	2.14	A_23_P139722
TNIP1	1.47	1.05	1.79	4.93	7.93	4.86	4.76	4.83	3.71	A_23_P30435
TPSAB1	0.72	0.51	0.79	0.93	0.67	0.73	0.63	0.64	324.80	A_23_P37702
WAS	1.64	1.59	0.47	0.68	2.04	1.18	0.31	0.93	4.33	A_23_P96331

Digestive function

Gene Symbol	DE	HG	hIO p0	hIO p2	Co-culture	IL-2	hAT-IO	hAT-IO+ NAM	hSI	Agilent Probe ID
CTRB2	1.08	1.96	2.04	1.33	0.84	1.13	0.49	0.57	4.37	A_32_P86150
ADA	1.21	1.02	0.82	0.76	0.16	0.37	0.12	0.11	2.24	A_23_P210482
MEP1B	0.87	0.62	2.01	1.13	6.73	1.63	19.24	9.61	296.45	A_23_P78353
MGAM	1.11	0.47	1.47	0.99	2.75	3.63	5.80	0.70	112.12	A_23_P42897
OAT	1.68	0.94	0.53	0.75	1.73	1.01	0.49	0.55	5.31	A_23_P98092
FABP2	0.87	1.47	0.96	1.13	84.76	9.21	42.19	31.95	184.36	A_23_P391711
GLB1	0.54	0.35	0.28	1.37	1.12	0.81	0.58	0.68	0.29	A_23_P61531
LCT	1.71	0.43	0.66	3.62	39.10	35.73	0.60	0.52	148.57	A_23_P79217
ASS1	0.84	0.80	0.23	0.24	0.72	0.33	2.01	2.90	0.32	A_33_P3234580
SI	0.87	0.62	0.96	21.58	30.63	49.22	137.40	64.75	1028.93	A_32_P302205
TREH	0.90	1.20	0.53	0.81	25.01	4.63	6.68	2.68	29.30	A_23_P104819
UGT1A8	0.87	0.70	0.96	1.13	115.31	5.45	611.20	458.83	277.52	A_33_P3302075
DPP4	1.52	1.30	2.68	4.39	51.96	23.16	4.12	6.97	9.20	A_33_P3287223
GIP	1.96	1.27	1.48	0.87	0.89	0.72	0.25	0.21	358.67	A_23_P141459

Intestinal markers

Gene Symbol	DE	HG	hIO p0	hIO p2	Co-culture	IL-2	hAT-IO	hAT-IO+ NAM	hSI	Agilent Probe ID
ALPI	0.70	0.93	0.43	1.28	3.18	1.40	1.66	0.78	81.79	A_23_P337658
BMI1	2.47	2.09	2.30	2.12	2.08	2.16	1.19	1.41	2.68	A_23_P314115
CA1	0.98	1.04	0.35	0.23	26.41	0.77	250.75	159.84	4.14	A_23_P168916
CDX2	0.93	94.32	117.81	432.88	3337.73	774.35	3171.39	2166.08	2288.92	A_33_P3304501
CFTR	0.75	0.17	0.24	0.56	5.02	0.87	5.32	2.52	6.63	A_23_P215720
CHGA	0.39	0.14	1.54	1.82	12.27	2.91	1.22	0.02	5.98	A_33_P3293164
FABP2	0.87	1.47	0.96	1.13	84.76	9.21	42.19	31.95	184.36	A_23_P391711
GUCY2C	0.89	0.16	0.09	1.09	71.66	20.85	27.87	25.23	35.29	A_23_P76312
HNF4A	0.87	0.72	0.96	3.65	20.17	10.44	10.72	13.04	10.17	A_23_P28761
ISX	0.87	6.05	127.70	713.29	6002.84	1811.29	5304.94	5321.55	1978.09	A_32_P217140
KRT20	0.89	0.81	0.67	15.30	1682.46	627.25	2354.93	2924.95	954.82	A_23_P66854
LGR5	46.18	53.47	9.29	42.75	20.12	13.82	1.44	0.75	9.12	A_23_P98974
MGAM	1.11	0.47	1.47	0.99	2.75	3.63	5.80	0.70	112.12	A_23_P42897
MUC13	0.89	0.93	0.96	35.76	1897.41	342.02	611.75	529.60	207.55	A_23_P155236
MUC2	0.68	0.60	0.27	0.38	2.65	1.35	1.38	0.47	35.52	A_33_P3412384
OLFM4	0.87	0.62	68.63	235.17	637.98	1123.73	17756.77	11462.02	47084.08	A_24_P181254
SOX9	3.52	2.51	16.46	35.02	27.58	24.63	11.60	11.95	19.00	A_23_P26847
TFF3	0.21	1.19	14.39	1339.10	1014.89	1076.05	755.82	704.06	548.75	A_23_P393099
VIL1	0.60	0.93	0.75	7.09	39.74	18.14	32.87	39.59	28.93	A_23_P16866

Color Index

The ratios are color-coded, as indicated by the color index bar. Red: Upregulated genes compared to undifferentiated control; blue: downregulated genes compared to undifferentiated control.

Fold	Color
>10	
>7.5	
>5	
>3	
>2	
>1.75	
>1.5	
>1.25	
1	
<0.8	
<0.6666666	
<0.5714286	
<0.5	
<0.3333333	
<0.2	
<0.1333333	
<0.1	

Supplementary Table 2. Levels of cytokines secreted by Jurkat T cells in various conditions as assessed by ELISA.

Cytokine expression levels (pg/ml)						
Cytokines	Jurkat T medium	NS Jurkat T	Stimulated Jurkat T w/ PMA and CI	hIO medium	NS Jurkat T in hIO medium w/ Matrigel	NS Jurkat T in hIO medium w/o Matrigel
IL-2	ND	2.00 ± 0.01	1003.59 ± 14.08	ND	1.00 ± 0.34	1.00 ± 0.51
IL-8	ND	5.86 ± 2.46	160.38 ± 23.41	ND	4.29 ± 1.43	2.86 ± 0.70
TNFα	1.88 ± 0.78	8.75 ± 2.50	36.88 ± 6.87	1.88 ± 0.67	5.00 ± 0.58	3.13 ± 0.63
IL-22	2.38 ± 0.83	3.33 ± 2.381	4.29 ± 2.38	2.38 ± 0.85	2.86 ± 0.95	3.33 ± 1.44
IL-6	ND	ND	ND	ND	ND	ND
IL-1β	2.80 ± 2.00	2.20 ± 0.60	3.80 ± 0.60	5.00 ± 2.60	4.80 ± 0.80	4.40 ± 1.62
IL-11	7.22 ± 1.67	1.67 ± 0.67	8.33 ± 0.56	8.89 ± 4.41	6.11 ± 1.67	3.33 ± 0.47
EGF*	ND	ND	ND	93941.4 ± 411.76	64882.5 ± 1823.53	57706.1 ± 1705.88
OSM	7.36 ± 0.47	6.27 ± 0.31	13.53 ± 1.17	8.38 ± 0.08	9.31 ± 0.23	7.59 ± 0.54
IL-10	ND	ND	ND	ND	ND	ND

All data are represented as mean ± SEM.

NS, non-stimulated.

ND, not detectable release.

* Cytokine included in hIO medium at concentrations of 100 ng/ml.

Supplementary Table 3. RNAseq datasets downloaded from public databases.

Ample Label	Description	Source	Donor ID	Accession #
hFSI #1	Fetal Day 91, Small intestine	GEO Datasets	H-23914	GSM1059508
hFSI #2	Fetal Day 98, Small intestine	GEO Datasets	H-23964	GSM1059521
hFSI #3	Fetal Day 108, Small intestine	GEO Datasets	H-23769	GSM1059486
hFSI #4	Fetal Day 108, Small intestine	GEO Datasets	H-23887	GSM1059507
hFSI #5	Fetal Day 115, Small intestine	GEO Datasets	H-23808	GSM1059517
hFSI #6	Fetal Day 120, Small intestine	GEO Datasets	H-23941	GSM1059519
hSI Dist #1	Distal Small Intestine	EMBL-EBI ArrayExpress	V151	E-MTAB-1733 (small intestine_4a)
hSI Dist #2	Distal Small Intestine	EMBL-EBI ArrayExpress	V152	E-MTAB-1733 (small intestine_4b)
hSI Dist #3	Distal Small Intestine	EMBL-EBI ArrayExpress	V153	E-MTAB-1733 (small intestine_4c)
hSI Dist #4	Distal Small Intestine	EMBL-EBI ArrayExpress	V156	E-MTAB-1733 (small intestine_4d)

Supplementary Table 4. List of the primers used in this study.

Gene	Primer (Forward)	Primer (Reverse)
GAPDH	GAAGGTGAAGGTCGGAGTC	GAAGATGGTGATGGGATTTC
CDX2	CTGGAGCTGGAGAAGGAGTTTC	ATTTTAACCTGCCTCTCAGAGAGC
SOX9	GGAGAGCGAGGAGGACAAGTTC	TTGAAGATGGCGTTGGGGG
LYZ	AAAACCCCAGGAGCAGTTAAT	CAACCCTCTTTGCACAAGCT
VIL1	AGCCAGATCACTGCTGAGGT	TGGACAGGTGTTCCTCCTTC
CHGA	TGACCTCAACGATGCATTTC	CTGTCCTGGCTCTTCTGCTC
MUC2	TGTAGGCATCGCTCTTCTCA	GACACCATCTACCTCACCCG
ISX	CAGGAAGGAAGGAAGAGCAA	TGGGTAGTGGGTAAAGTGGAA
LGR5	TGCTCTTCACCAACTGCATC	CTCAGGCTCACCAGATCCTC
SI	GGTAAGGAGAAACCGGGAAG	GCACGTCGACCTATGGAAAT
VIM	AGAACGTGCAGGAGGCAGAAGAAT	TTCCATTTCACGCATCTGGCGTTC
OLFM4	ACCTTTCCCGTGGACAGAGT	TGGACATATTCCCTCACTTTGGA
DEFA5	CCTTTGCAGGAAATGGACTC	GGACTCACGGGTAGCACAAC
DEFA6	GCCTAGACACTGATGACCCC	GCATGCTGTATTGCGCCTC
KRT20	TGGCCTACACAAGCATCTGG	TAACTGGCTGCTGTAACGGG
SLC5A1	GTGCAGTCAGCACAAAGTGG	ATGCACATCCGGAATGGGTT
MUC13	CGGATGACTGCCTCAATGGT	AAAGACGCTCCCTTCTGCTC
CREB3L3	ATCTCCTGTTTGACCGGCAG	GTCGTCAGAGTCGGGGTTTG
IL-2Rα	TCTTCCCATCCCACATCCTC	TCTGCGGAAACCTCTCTTGC
IL-2Rβ	GGCTTTTGGCTTCATCATCT	CTTGTCCCTCTCCAGCACTT
IL-2Rγc	ACGGGAACCCAGGAGACAGG	AGCGGCTCCGAACACGAAAC
P-GP	GCCAAAGCCAAAATATCAGC	TTCCAATGTGTTCGGCATTA
B2m	CTGGTGCTTGTCTCACTGAC	GTTCAGTATGTTCGGCTTCC
Cdx2	GCAGTCCCTAGGAAGCCAAGTGA	CTCTCGGAGAGCCCAAGTGTG
Olfm4	TGGCCCTTGGAAGCTGTAGT	ACCTCCTTGGCCATAGCGAA
Lyz	GCCAAGGTCTACAATCGTTGTGAGTTG	CAGTCAGCCAGCTTGACACCACG
Dpp4	AGGATCACATCGACAGGAGAA	TTGTTTGGAGACCACCACAG
Defa5	ACTGAGGAGCAGCCAGGGGA	ACGCGTTCTCTTCTTTTGCAGCC
Defa6	CTCTCTGCCCTCGTCCTG	CCTGGCTGCTCCTCAGTTT
Krt20	CCTGCGAATTGACAATGCTA	CCTTGGAGATCAGCTTCCAC
Lct	TGTCCTAGCCTACAACCTCAAC	AGCGGTCTGTAATGGAAGCA
Slc5a1	CCACAAAGTGACCACTTCCA	GTGGTACCGTTGGAGGCTT
Muc13	TCCCTGGGGACATTAGCA	GGCTAGGGAGGGTTCCAA
Creb3l3	CCAGACCCTTTACCCATACAT	ATGGTTGGAGGTTAGGGTTCAG

Supplementary Table 5. List of antibodies used in this study.

Antibodies	Catalog No.	Company	Dilution
**Pluripotency markers**			
anti-OCT4	sc-9081	Santa Cruz	1:100 for IF
anti-NANOG	AF1997	R&D systems	1:40 for IF
anti-SSEA-3	MAB1434	Millipore	1:30 for IF
anti-SSEA-4	MAB1435	Millipore	1:30 for IF
anti-TRA-1-60	MAB4360	Millipore	1:100 for IF
anti-TRA-1-81	MAB4381	Millipore	1:100 for IF
***In vitro* differentiation markers**			
anti-TUJ1	PRB-435P	Covance	1:500 for IF
anti-NESTIN	MAB5326	Millipore	1:100 for IF
anti-FOXA2	07-633	Millipore	1:100 for IF
anti-SOX17	MAB1924	R&D systems	1:50 for IF
anti-DESMIN	AB907	Chemicon	1:50 for IF
anti-α-SMA	A5228	Sigma	1:500 for IF
**Intestinal organoid differentiation markers**			
anti-CDX2	ab15258	abcam	1:100 for IF
anti-KLF5	ab137676	abcam	1:100 for IF
anti-SOX9	sc-7314	Santa Cruz	1:50 for IF
anti-Villin	sc-7672	Santa Cruz	1:50 for IF
anti-Mucin2	sc-7314	Santa Cruz	1:50 for IF
anti-Chromogranin A	MA5-14536	Thermo Scientific	1:200 for IF
anti-Lysozyme	ab76784	abcam	1:200 for IF
anti-E-Cadherin	610182	BD Biosciences	1:200 for IF
anti-E-Cadherin	AF648	R&D systems	1:500 for IF
**Intestine maturation markers**			

anti-alpha 5 Defensin	ab90802	abcam	1:50 for IF
anti-OLFM4	ab85046	abcam	1:100 for IF
anti-MUC13	ab124654	abcam	1:100 for IF
anti-Cytokeratin 20	ab76126	abcam	1:400 for IF
anti-Ki67	AB9296	Chemicon	1:100 for IF
**Intestinal transporter markers**			
anti-SI (Sucrase-isomaltase)	HPA011897	Sigma	1:100 for IF
anti-PEPT1	sc-20653	Santa Cruz	1:100 for IF
anti-MDR-1	MAB4120	Chemicon	1:100 for IF
**Vasculature markers**			
anti-hCD31	MA5-15336	Thermo Scientific	1:400 for IF
anti-PECAM-1	sc-8306	Santa Cruz	1:50 for IF
anti-MECA-32	NB100-77668	Novus Biologicals	1:400 for IF
anti-VEGF	sc-152	Santa Cruz	1:50 for IF
**STAT3 signaling markers**			
anti-STAT3	#9132	Cell Signaling	1:2000 for WB
anti-phospho-STAT3(Tyr707)	#9131S	Cell Signaling	1:2000 for WB
anti-AKT	#9272S	Cell Signaling	1:1000 for WB
anti-phospho-AKT(Ser473)	#9271S	Cell Signaling	1:1000 for WB
anti-P70-S6-kinase	#2708	Cell Signaling	1:1000 for WB
anti-phospho-P70-S6-kinase(Thr389)	#9205	Cell Signaling	1:1000 for WB
anti-β-Actin	sc-81178	Santa Cruz	1:2000 for WB
**IL-2 receptor antibody**			
anti-IL-2 receptor α	ab61777	abcam	1:2000 for WB
anti-IL-2 receptor β	ab197934	abcam	1:2000 for WB
anti-IL-2 receptor γ	ab180698	abcam	1:2000 for WB
**IL-2 receptor blocker**			
anti-IL-2 receptor β	AF-224-NA	R&D systems	3ug/ml for cell treatment
anti-IL-2 receptor γ	MAB2842	R&D systems	100ng/ml for cell treatment

Lamina propria Lymphocytes markers			
PerCP Hamster IgG1, κ Isotype Contro	553975	BD bioscience	1:200 for FACS
APC Rat IgG2b, κ Isotype Control	556924	BD bioscience	1:200 for FACS
PerCP Hamster Anti-Mouse CD3e	561089	BD bioscience	1:200 for FACS
APC Rat Anti-Mouse IL-2	562041	BD bioscience	1:200 for FACS
FITC Rat Anti-Mouse CD4	561831	BD bioscience	1:500 for FACS
PE Rat Anti-Mouse CD4	561829	BD bioscience	1:200 for FACS
FITC Rat Anti-Mouse CD8a	561966	BD bioscience	1:500 for FACS
PE Rat Anti-Mouse CD45	561087	BD bioscience	1:200 for FACS

*IF: Immunofluorescence
*WB: Western blotting

Supplementary Table 6. Probes used for FISH analysis.

RNAscope® Probe Hs-DEFA5 (Cat. No. 423981)

Specifications	
Gene	DEFA5
Species*	Human
Species (common)	Human
Entrez Gene ID	1670
Gene Alias	DEF5
Accession No	NM_021010.1
Target Region	2 - 430
No. of Pairs	8

RNAscope® Hs-OLFM4 probes

Specifications	
Gene	OLFM4
Species*	Human
Species (common)	Human
Entrez Gene ID	10562
Gene Alias	GC1
Accession No	NM_006418.4
Target Region	1111 - 2222
No. of Pairs	20

RNAscope® Positive Control Probe Hs-PPIB (Cat. No. 313901)

Specifications	
Gene	PPIB
Species*	Human
Species (common)	Human
Entrez Gene ID	5479
Gene Alias	CYP-S1
Accession No	NM_000942.4
Target Region	139 - 989
No. of Pairs	16

RNAscope® Negative Control Probe Hs-DapB (Cat. No. 310043)

Specifications	
Gene	DapB
Species*	Other
Species (common)	Unclassified
Entrez Gene ID	N/A
Gene Alias	N/A
Accession No	EF191515
Target Region	414 - 862
No. of Pairs	10
